# Pathogenic Mechanism of a Highly Virulent Infectious Hematopoietic Necrosis Virus in Head Kidney of Rainbow Trout (*Oncorhynchus mykiss*) Analyzed by RNA-Seq Transcriptome Profiling

**DOI:** 10.3390/v14050859

**Published:** 2022-04-21

**Authors:** Jinwoo Kim, Miyoung Cho, Jongwon Lim, Hyeseong Choi, Suhee Hong

**Affiliations:** 1 Department of Marine Biotechnology, Gangneung-Wonju National University, Gangneung 25457, Korea; zinu@gwnu.ac.kr (J.K.); myobong@gwnu.ac.kr (J.L.); 2 Pathology Research Division, National Institute of Fisheries Science, Busan 46083, Korea; mycho69@korea.kr (M.C.); choihs@korea.kr (H.C.)

**Keywords:** IHNV, pathogenesis, transcriptome, rainbow trout

## Abstract

Infectious hematopoietic necrosis virus (IHNV) is a pathogen that causes high rates of mortality in salmonid fishes. Therefore, an RNA-seq-based transcriptome analysis was performed in the head kidney of rainbow trout infected with a highly virulent IHNV strain to understand the pathogenesis of and defense strategies for IHNV infection in rainbow trout. The results showed that the numbers of DEGs were 618, 2626, and 774 (control vs. IHNV) on days 1, 3, and 5, respectively. Furthermore, the enrichment analysis of gene ontology (GO) annotations to classify DEGs showed that GO terms considerably associated with DEGs were gluconeogenesis, inflammatory response, and cell adhesion in the Biological Process (BP) category, apical plasma membrane, extracellular matrix (ECM) in the Cellular Component category, and transporter activity, integrin binding, and protein homodimerization activity in the Molecular Function category, on days 1, 3, and 5, respectively. Notably, GO terms in the BP category, including the negative regulation of type I interferon production and positive regulation of interleukin-1β secretion, were commonly identified at all time points. In the Kyoto Encyclopedia of Genes and Genomes (KEGG) pathway analysis, complement and coagulation cascades were commonly identified at all time points. Importantly, the widely recognized GO terms and KEGG pathways extensively linked to DEGs were related to energy metabolism on day 1, the immune response on day 3, and cell proliferation on day 5. Furthermore, protein–protein interaction networks and centrality analysis showed that the metabolism and signaling transduction pathways were majorly upregulated. Conclusively, the virulent IHNV infection drives pathogenesis by activating the metabolic energy pathway for energy use for viral replication, facilitating necrosis through autophagy, and causing a shutoff response of the host immune system through the downregulation of type I IFN at the initial stage of infection.

## 1. Introduction

Infectious hematopoietic necrosis virus (IHNV) is an economically significant pathogen that causes clinical disease and mortality in many salmonid species, such as Atlantic salmon (*Salmo salar*), chinook salmon (*Oncorhynchus tshawytscha*), coho salmon (Oncorhynchus kisutch), and rainbow trout (*Oncorhynchus mykiss*) [1]. The IHNV is an enveloped single-stranded RNA (negative-sense) virus that belongs to the family Rhabdoviriae [1], now predominant in Asian and European countries through the transportation of IHNV-contaminated fish or fish eggs from the Pacific Northwest of North America [1,2,3]. Additionally, the Korean isolates of IHNV are genetically similar to the Japanese isolates, further grouped based on the phylogenetic analysis of glycoprotein (G) and non-virion protein (NV) gene sequences into IHNV-Shizuoka and IHNV-Nagano genogroups [4,5,6]. Although previous studies classified the IHNV-Shizuoka genotype as more virulent than the IHNV-Nagano genotype in salmonid fishes [5,7], it is difficult to determine its virulence based on its genotype persistence, since virulence was tested only in a few isolates. Certainly, we have reported the highly pathogenic Nagano lineage strains [8]. Therefore, no plausible relationship between genotype and pathogenicity in IHNV-Shizuoka and IHNV-Nagano strains exists so far.

Additionally, we previously reported a transcriptome analysis of rainbow trout infected by a low virulent IHNV strain of the Nagano genotype [8]. This strain’s infection dynamically altered the transcriptome profiles in the head kidney of rainbow trout, inducing a defense mechanism by regulating the immune and inflammatory pathways through pattern recognition receptor (PRR) signaling at the initial stage of day 1 post-infection. Furthermore, the downregulated pathways involved in extracellular matrix formation and focal adhesion on day 5 revealed obvious wound healing failure, particularly essential in the IHNV infection pathogenesis. However, the molecular mechanisms of the pathogenicity are different in a highly virulent strain.

In a previous study, greater host cell shutoff responses were observed in a microarray analysis by a more virulent IHNV M genotype virus than by a less virulent U genotype virus in rainbow trout [9]. Host cell shutoff responses by the IHNV M genotype virus facilitated subversion of the host cell transcriptional machinery and enhanced viral replication and manipulated the transcriptional and translational machinery [9]. Many members of RNA virus families, such as picornaviruses and rhabdoviruses, are known to inhibit host RNA synthesis by blocking transcription of all three host RNA polymerases (RNAPs) [10]. The inhibition of host RNAPII is known to be caused by inactivation of transcription initiation factors in these viruses [10]. The M protein of the IHNV induced the downregulation of host transcription in vitro and programmed apoptosis [11]. Additionally, Cho et al. demonstrated through RNA-seq analysis that viral hemorrhagic septicemia virus (VHSV) caused necrosis in host cells by inhibiting metabolic functions such as ATP synthesis and the antioxidant system in olive flounder (*Paralichthys olivaceus*) [12]. Previous studies also showed a considerable increase in the glycolytic intermediates infected with the human cytomegalovirus (HCMV) [13]. However, human foreskin fibroblast (HFF) cells infected with the vaccinia virus did not induce glycolysis intermediates; the virus replicated, forming virions to remove glucose from the medium [14]. Although several viruses seem to use glycolysis, it is not universal.

Therefore, transcriptome profiles were investigated in rainbow trout to identify the pathogenesis of virulent IHNV infection. As a result, we confirmed the pathogenicity of the IHNV RtCc0517c isolate in rainbow trout. We also performed RNA-seq and real-time quantitative polymerase chain reaction (Q-PCR) in the head kidney at 1, 3, and 5 days after the viral challenge.

## 2. Materials and Methods

### 2.1. Ethics Statement

The Institutional Animal Care Use Committee of the Gangneung–Wonju National University approved all fish handling and experimental procedures. All experiments were performed following the relevant guidelines and regulations.

### 2.2. Preparation of Fish

Healthy rainbow trout (body weight 37 ± 2 g) were obtained from an aquaculture farm in Pyeongchang, Korea. Then, the fish were acclimatized in a 50 L aquarium at 12 ± 1 °C for 2 weeks and fed daily (1.5% body weight) with a commercial pelleted diet (Woosung Feed Co., Hongseong, Korea). After acclimation, IHNV infection was checked through reverse transcription-PCR using a primer pair designed to amplify the conserved region of G and NV genes of IHNV in the kidney [5]. Notably, IHNV infection was not observed in the experimental fish (data not shown).

### 2.3. Preparation of Virus

The IHNV RtCc0517c strain was isolated from rainbow trout at fish farms during the Korean National Surveillance Program by Kim et al. [7] and identified as possessing the IHNV-Nagano genotype. The mortality in the fish farm was observed to be 80%. First, 50 mg of kidneys from infected fish were homogenized in PBS and inoculated onto a monolayer of the epithelioma papulosum cyprini (EPC, ATCC CRL-2872) cell line cultured at 20 °C in Eagle’s minimum essential medium (EMEM, ATCC, Manassas, VA, USA) supplemented with 10% fetal bovine serum (FBS, Gibco, Waltham, MA, USA) and 1% antibiotic–antimycotic solution (Gibco). Next, the inoculated cells were incubated in a 25 cm^2^ tissue culture flask (Corning Inc., Corning, NY, USA) at 15 °C and observed daily for 10 days. At this point, 500 μL of supernatant was re-inoculated into fresh cells for a further 10 days as a blind passage. After the appearance of a cytopathic effect (CPE), RT-PCR and sequencing were performed for confirmation of IHNV.

### 2.4. Virulence Test by Intraperitoneal (i.p.) Challenge

To confirm the pathogenicity of IHNV RtCc0517c, 40 fish (average weight of 37 ± 2 g) were separated into 2 groups in 27 L rectangular tanks with steady flowing water at 12 ± 1 °C. Next, the fish were anesthetized using 3 mg/mL of 2-phenoxyethanol and i.p. injected with 100 μL of PBS or 10^4^ plaque-forming units (PFU)/fish of live IHNV RtCc0517c. Lastly, the fish were observed daily to determine the quantity of dead fish over 30 days and obtain the final cumulative percent mortality (CPM).

Additionally, 60 fish (average weight of 37 ± 2 g) were placed into 2 groups and divided into 6 27 L rectangular tanks with 10 fish in each tank. Next, the fish were anesthetized using 3 mg/mL of 2-phenoxyethanol and i.p. injected with PBS or 104 PFU/fish of viral culture. The fish were sacrificed by anesthetization using 3 mg/mL of 2-phenoxyethanol and cutting of the spinal cord on days 1, 3, and 5. Subsequently, the head kidney was removed aseptically, quickly frozen in liquid nitrogen, and stored at −70 °C until RNA isolation.

### 2.5. RNA Isolation and Sequencing

First, the frozen head kidney tissue was crushed in liquid nitrogen using mortars and pestles; then, total RNA was extracted using TRIzol (Invitrogen, Waltham, MA, USA), based on the manufacturer’s instructions. Next, RNA degradation and concentrations were measured after dissolving in RNase-free water (Gibco) using electrophoresis on 1.5% (*w*/*v*) agarose gels and NanoDrop (Effendorf), respectively, and finally stored at −80 °C until use.

For the RNA sequencing, 3 samples in a group on days 1, 3, and 5 were examined to estimate the quality and quantity of total RNA using Quant-IT RiboGreen (Invitrogen, #R11490) and total RNA integrity by being run on the TapeStation RNA screen tape (Agilent, #5067-5576). Next, 2 of 3 samples in a group at each time point of high-quality RNA preparations with RIN greater than 7.0 were chosen and used to construct the RNA library. Similarly, a library was independently prepared from 1 μg of total RNA for each sample using an Illumina TruSeq Stranded mRNA Sample Prep Kit (Illumina, San Diego, CA, USA, #RS-122-2101). The poly-A-containing mRNA molecules were subsequently purified using poly-T-attached magnetic beads, fragmented into small pieces using divalent cations under increased temperature, and copied into first strand cDNA using SuperScript II reverse transcriptase (Invitrogen, #18064014) and random primers. Additionally, the second strand cDNA synthesis was performed using DNA polymerase I, RNase H, and dUTP that underwent an end repair process, including a single “A” base and ligation of the adapters. Finally, the products were PCR purified and enriched to create the final cDNA library. The libraries were computed using KAPA Library Quantification Kits for Illumina Sequencing platforms following the RT-qPCR quantification protocol guide (KAPA BIOSYSTEMS, #KK4854) and qualified by the TapeStation D1000 ScreenTape (Agilent Technologies, #5067-5582). Lastly, indexed libraries were submitted to an Illumina NovaSeq analysis (Illumina, San Diego, CA, USA), and Macrogen Incorporated performed the paired-end (2 × 100 bp) sequencing.

### 2.6. Data Analysis

#### 2.6.1. Transcriptome Annotation

The RNA-seq data were polished by discarding low-quality reads containing the unknown bases or those whose lengths were lower than 20 nucleotides after removing the adaptors and low-quality bases. The base quality and duplication level were visually confirmed using FastQC software [15] to quality check the Fastq format files. Next, clean reads were generated from the raw reads by removing low-quality reads, those containing adapters and poly-N using RNA-QC-chain [16], and then mapped onto the *Oncorhynchus mykiss* reference genome (accession no. GCF_002163495.1) using TopHat v 2.0.13 [17]. After mapping clean reads onto the reference genome (accession no. GCF_002163495.1), we enumerated the quantities and percentages of the uniquely mapped reads. The known and new copies were identified in the TopHat sort results using Cufflinks v 2.1.1 [18] with optional multi-read correction, frag-bias-correct, and default parameters. The expression levels were assessed by calculating the expected number of fragments per kilobase of transcript sequence per million base pairs (FPKM) [18]. The correlation between each sample was also calculated using the expression values of FPKM. Moreover, gene expression levels correlated with samples were assessed to verify the reliability of an experiment, the square of the Pearson correlation coefficient (R^2^) shown to be over 0.9, meeting the prerequisite for differential expression analysis. The differentially expressed genes (DEGs) were also assigned to different functional categories to facilitate the global gene expression analysis using InterProScan [19].

#### 2.6.2. DEG Analysis

DEGs were calculated using Cuffdiff [20]. Additionally, multi-read-correction and frag-bias-correct options were added for accurate analysis. The genes with FPKM ratios greater than two folds or lower than 0.05 folds were considered up- or downregulated, respectively, and then defined as DEGs.

#### 2.6.3. Gene Ontology (GO) Analysis of DEGs

In analyzing the DEGs using GO, gene characteristics were classified into three categories using the DAVID Bioinformatics resources 6.8 software: Biological Process (BP), Cellular Component (CC), and Molecular Function (MF). Additionally, the GO information was screened and annotated according to the human database, since rainbow trout GO information was not provided in the databases. Furthermore, to clarify the biological functions of all identified DEGs, the cutoff of GO terms was *p* < 0.05 and fold enrichment >2.

#### 2.6.4. KEGG Pathway Analysis of DEGs

Additionally, DEGs were evaluated for Kyoto Encyclopedia of Genes and Genomes (KEGG) pathway enrichment using the DAVID Bioinformatics resources 6.8 software according to the pathways and relationships verified in the human system. Moreover, since the verified KEGG interactions and pathways at the single species level are not meant to accommodate multi-species comparisons, we ignored the terms related to proteins of non-model species, considering only those mapped in the human species model. In clarifying the biological pathways of all DEGs regulated by IHNV infection, the cutoff of KEGG terms was *p* < 0.05 and fold enrichment >2.2.6.5. Functional and Protein–Protein Interaction (PPI) Analysis of DEGs

PPI networks were analyzed using the STRING (http://string-db.org/, accessed on 5 June 2020) database, including the direct and indirect associations of proteins, to understand the functions of the DEGs. After analyzing the result from STRING analysis and the expression change information for each DEG, the network figure was drawn for the selected DEGs (connected with one or more DEGs) using Cytoscape 3.7.1 software.

### 2.7. cDNA Synthesis and Quantitative PCR (Q-PCR) Analysis

Total RNA was extracted from 8 fish in a group at each time point. Initially, the first strand cDNA was synthesized using a RevertAid H Minus First Strand cDNA synthesis kit (Thermo Scientific, Waltham, MA, USA), following the manufacturer’s instructions. Briefly, 3 μg of total RNA in 12μL of DEPC-treated water was incubated with 1 μL of oligo (dT)18 primer (100 μM; Thermo) at 65 °C for 5 min and reverse transcribed by adding a mixture of 0.5 μL of the RevertAid reverse transcriptase (100 U/mL; Thermo), 4 μL of 5× first strand buffer (Thermo) containing 250 mM Tris-HCl (pH 8.3), 250 mM KCl, 20 mM MgCl_2_ and 50 mM DTT, 0.5 μL of RiboLock RNase Inhibitor (20 U/mL; Thermo), and 2 μL of 10 mM dinucleoside triphosphate (dNTP) mix (Thermo) at 42 °C for 2 h. Lastly, the reaction was terminated by heating to 70 °C for 10 min, and 380 μL of TE buffer was added to make up the final volume to 400 μL.

Q-PCR was carried out to authenticate the RNA-seq results. Here, 12 genes, including IL-18, IFN2, IL-8, IRF9, IL-6, MT-ATP8, TNF-α, IL-1β, COX2, TP53, PTK2, and RAC1, were selected for the RT-qPCR assay since they are differentially regulated after infection in the KEGG pathway analysis. Table S1 shows the primers for these genes.

Q-PCR was performed in a 20 μL reaction containing 10 μL of SYBR Green Real-time PCR Master Mix (Takara, Japan), 0.4 mM of each forward- and reverse- primer, and 4 μL of cDNA according to the following protocol: 60 s at 95 °C; the template was amplified for 40 cycles of denaturation for 15 s at 95 °C, annealing, and extension for 1 min at 60 °C using the LC96 real-time thermocycler (Roche) [21]. Each sample was analyzed in duplicate combined with a serial dilution of references for the absolute quantification analysis, and the transcript level was calculated using the integrated software previously described [22]. Data normalization was performed using a housekeeping gene, i.e., elongation factor (EF)-1α. EF-1α is one of the reference genes most used to reduce possible error generated in quantifying genes and normalizing Q-PCR [23]. Lastly, fold change was calculated after normalization to EF-1α expression level by dividing the ratio of EF-1 α by a negative control sample at each time point. Q-PCR data were expressed as means ± standard error and were compared via an unpaired sample *t*-test using SPSS 23.0 for Windows (SPSS Inc., Chicago, IL, USA).

## 3. Results

### 3.1. Mortality in the IHNV Challenge Test

**Figure 1:**
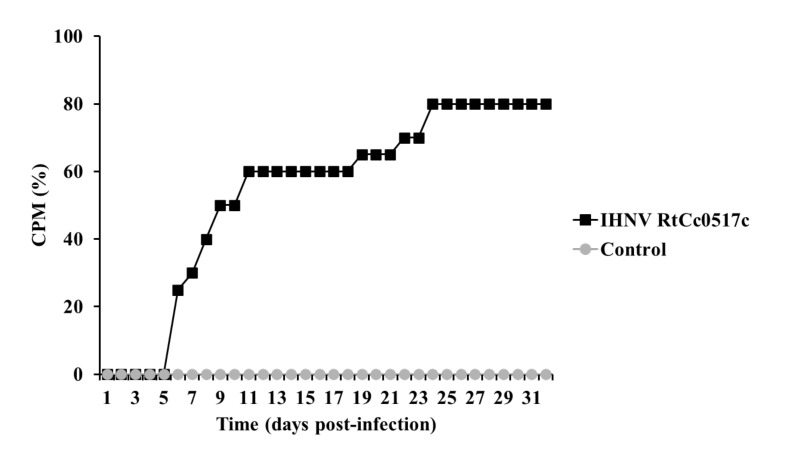
Cumulative percent mortality (CPM) of rainbow trout challenged by intraperitoneal injection with IHNV. Forty rainbow trout were divided into two groups and challenged with PBS or the IHNV RtCc0517c strain (10^4^ PFU/fish). Mortality was recorded daily for 30 days.

Fish infected with the IHNV RtCc0517c strain started dying from day 5, and final CPM reached 85% on day 30, whereas no mortality was observed in the control group for 30 days (Figure 1).

### 3.2. Overview of DEGs

RNA-seq obtained more than 7Gb data for each sample and an average of 90,803,874 raw reads. After filtering, an average of more than 88,367,158 clean reads were generated for all samples (Figure S1B, Table S2). The total length of the reads averaged 9.17 × 10^9^ base pairs for all samples (Figure S1A, Table S2). Q20 and Q30 percentages (the percentage of sequences with a sequencing error rate lower than 1% and 0.1%, respectively) were over 98% and 95%, respectively, for all samples (Figure S1D, Table S2). The GC percentage was over 48% for all samples (Figure S1C, Table S2). Furthermore, all the high-quality reads were deposited in the National Center for Biotechnology Information Sequence Read Archive and can be accessed under the accession number (accession no. PRJNA612101). During annotation, above 80% of clean reads were mapped to the rainbow trout reference genome (accession no. GCF_002163495.1) (Table S3), confirming that the alignment of the RNA sample progressed well. Additionally, gene expression levels were determined by calculating the number of unambiguous reads for each gene normalized using the FPKM method [18], which obtained the number of DEGs for each time point, as shown in Table 1.

### 3.3. Differential Expression Profile

The Pearson correlation coefficient square (R2) was over 0.9, meeting a requirement for differential expression analysis (Figure S2). The total number of DEGs between the control group versus the IHNV-infected group was highest on day 3, as the numbers of DEGs between the control and IHNV-infected groups were 618, 2626, and 774 on days 1, 3, and 5, respectively (Table 1).

### 3.4. Gene Ontology (GO) Analysis

In GO enrichment analysis, the numbers of GO terms significantly enriched in DEGs were 115, 341, and 118 on days 1, 3, and 5, respectively (Table S4). GO terms commonly identified in all time points were “negative regulation of type I IFN production”, “positive regulation of smooth muscle cell proliferation”, “positive regulation of interleukin-1β secretion”, “defense response to virus”, “platelet degranulation”, “response to virus”, “positive regulation of VEGF production”, in the BP category, and “platelet alpha granule lumen”, “basolateral plasma membrane”, and “basal plasma membrane”, in the CC category (Table 2). GO terms considerably associated with DEGs were gluconeogenesis, inflammatory response, and cell adhesion, in BP; apical plasma membrane, extracellular matrix (ECM), and ECM, in CC; and transporter activity, integrin binding, and protein homodimerization activity, in MF, on days 1, 3, and 5, respectively (Tables S5–S7). Additionally, GO BP terms majorly associated with DEGs related to energy metabolism (gluconeogenesis, transmembrane transport, cellular response to hypoxia) on day 1, immune response (inflammatory response, leukocyte migration, cytokine-mediated signaling pathway) on day 3, and cell proliferation (cell adhesion, extracellular matrix organization, positive regulation of smooth muscle cell proliferation, ERBB2 signaling pathway) on day 5, respectively (Table 3).

### 3.5. Kyoto Encyclopedia of Genes and Genomes (KEGG) Analysis

The enrichment analysis for KEGG pathways identified common pathway terms of complement and coagulation cascades at all time points. These included glycine, serine, and threonine metabolism and proximal tubule bicarbonate reclamation on days 1 and 3; cell adhesion molecules, malaria, and measles on days 3 and 5; while ABC transporters on days 1 and 5 were significantly enriched in DEGs between control and IHNV (Table 4). The DEGs between control and IHNV were primarily associated with KEGG terms related to metabolism (bile secretion, PPAR signaling pathway, carbon metabolism, biosynthesis of antibiotics, histidine metabolism, biosynthesis of amino acids, glyoxylate and dicarboxylate metabolism, metabolic pathway, arginine and proline metabolism, adipocytokine signaling pathway, AMP-activated protein kinase (AMPK) signaling pathway, mineral adsorption, glycolysis/gluconeogenesis, β-alanine metabolism) on day 1, signal transduction and immune system (cytokine–cytokine receptor interaction, cell adhesion molecules, NF-κB signaling pathway, Toll-like receptor signaling pathway, TNF signaling pathway, RIG-I-like receptor signaling pathway, NOD-like receptor signaling pathway, cytosolic DNA-sensing pathway, Jak–STAT signaling pathway) on day 3, and signaling and immune system (cell adhesion molecules, ECM–receptor interaction) (Table 5).

### 3.6. Protein–Protein Interaction (PPI) Analysis of DEGs

**Figure 2:**
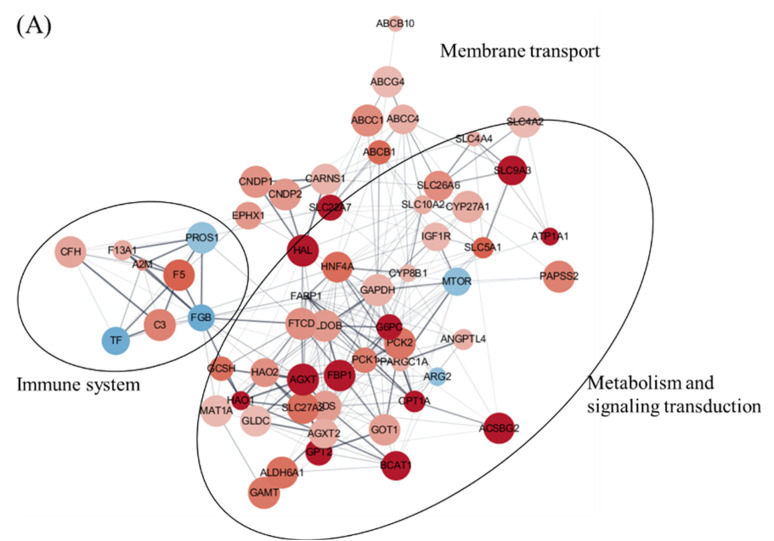

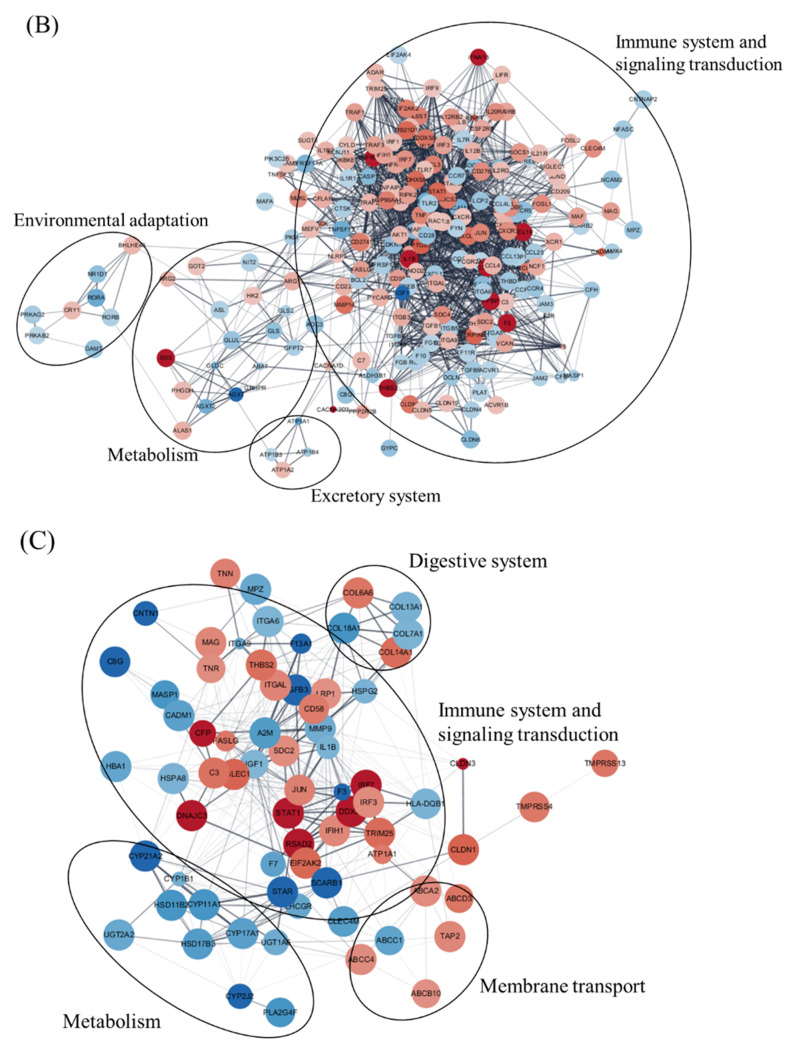
Protein–protein interaction network analysis of DEGs. Control vs. IHNV. The STRING database analyzed the protein–protein interaction network based on the proteins matching the selected DEGs on days 1 (**A**), 3 (**B**), and 5 (**C**). The protein interaction relationship of DEGs existing in the string database was selected for the network formation. The red color indicates upregulated genes; the blue color indicates downregulated genes. Node sizes are proportional to *p*-values.

Additionally, to further understand the biological relevance of DEGs, a PPI network analysis of DEGs was carried out using the STRING database. The PPI network analysis of DEGs identified 279, 241, and 165 interactional relationships among 54, 73, and 50 genes within the DEGs on days 1, 3, and 5, respectively (Figure 2, Table 6). In the PPI analysis, genes in the metabolism and signaling transduction network were upregulated with the highest centrality of the glyceraldehyde 3-phosphate dehydrogenase (GAPDH) gene followed by phosphoenolpyruvate carboxykinase 1 (PCK1), alanine-glyoxylate and serine-pyruvate aminotransferase (AGXT), hepatocyte nuclear factor 4 alpha (HNF4A), glucose 6-phosphatase alpha (G6PC), phosphoenolpyruvate carboxykinase 2 (PCK2), fatty acid-binding protein 1 (FABP1), aldolase fructose-bisphosphate B (ALDOB), formimidoyltransferase cyclodeaminase (FTCD), and solute carrier family 5 member 1 (SLC5A1) (Figure 2A, Table 6). Furthermore, on days 3 and 5, the immune system and signaling transduction network mainly appeared and were upregulated, with the highest centrality of TNF and MMP genes, respectively; however, the metabolism-related genes were downregulated (Figure 2B,C, Table 6). 

### 3.7. Analysis of DEGs of Immune- and Metabolism-Related Genes

**Figure 3:**
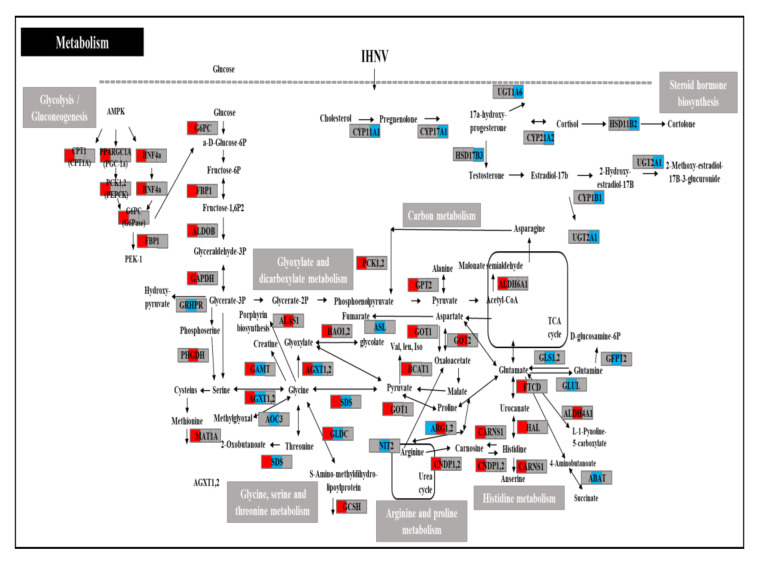
Presentation of putative metabolism-related pathways on days 1, 3, and 5 in the IHNV-infected group. DEGs regulated by the IHNV infection are shown in red (upregulated) or blue (downregulated). The box is divided into three spaces, indicating the up- and downregulated genes on days 1, 3, and 5. The black arrows show the activation and regulatory responses of downstream pathways.

**Figure 4:**
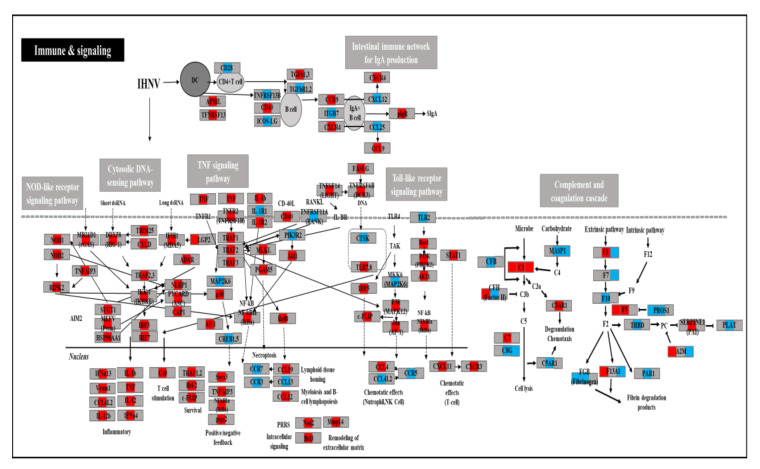
Presentation of putative immune pathways on days 1, 3, and 5 in the IHNV-infected group. DEGs regulated by the IHNV are shown in red (upregulated) or blue (downregulated). The box is divided into three spaces, indicating the up- and downregulated genes on days 1, 3, and 5. The black arrows show the activation and regulatory responses of downstream pathways.

The results showed that out of 17 on day 1, 23 on day 3, and 12 on day 5 mapped in the KEGG database, 13 metabolic-related KEGG pathways and 12 immune- and signaling-related KEGG pathways were detected. Tables S8 and S9 shows the DEGs for the 25 metabolic- or immune- and signaling-related KEGG pathways. Additionally, out of 238 DEGs, 68 annotated genes were identified as being involved in metabolic-related KEGG pathways in carbon metabolism; biosynthesis of antibiotics; histidine metabolism; biosynthesis of amino acids; glyoxylate and dicarboxylate metabolism; arginine and proline metabolism; glycolysis/gluconeogenesis; β-alanine metabolism; glycine, serine, and threonine metabolism; AMPK signaling pathway; alanine, aspartate, and glutamate metabolism; arginine biosynthesis; steroid hormone biosynthesis; and steroid hormone biosynthesis (Figure 3). Additionally, 170 annotated DEGs were identified as being involved in immune and signaling pathways in complement and coagulation cascades, cytokine–cytokine receptor interactions, cell adhesion molecules (CAMs), the Toll-like receptor signaling pathway, the TNF signaling pathway, the NF-kappa B signaling pathway, complement and coagulation cascades, the RIG-I-like receptor signaling pathway, the cytosolic DNA-sensing pathway, the NOD-like receptor signaling pathway, the intestinal immune network for IgA production, and ECM–receptor interactions (Figure 4).

### 3.8. Verification of RNA-Seq Data by RT-qPCR

**Figure 5:**
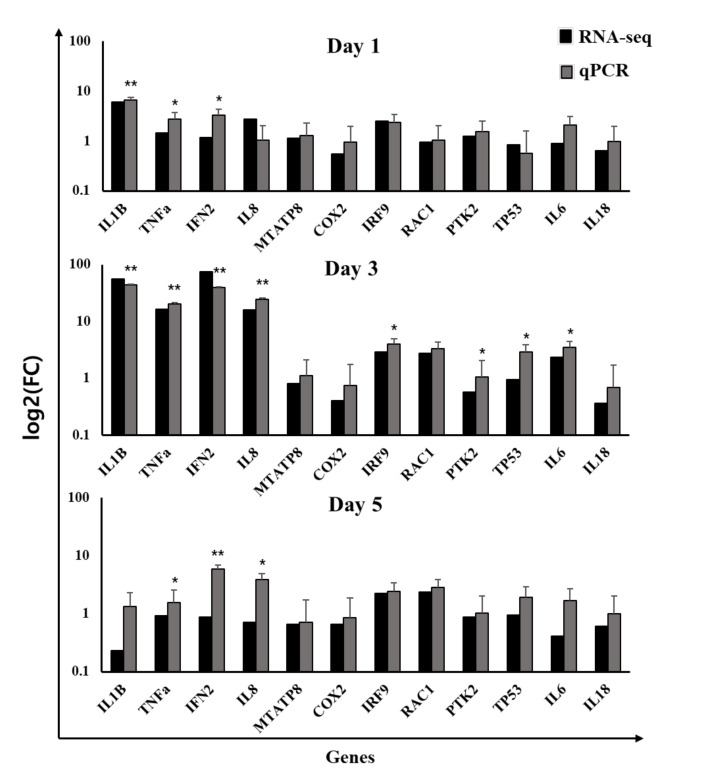
Expression level of 12 randomly selected genes validated by Q-PCR. The EF-1α gene was used as an internal control, and each gene’s relative quantity of gene expression (fold change) was calculated comparatively. The asterisks indicate the level of significance based on an unpaired *t*-test (*, *p* < 0.05; **, *p* < 0.00; N = 8). IL-1β, interleukin-1beta; TNFα, tumor necrosis factor-alpha; IFN2, Type I IFN 2; IL-8, interleukin-8; MT-ATP8, ATP synthase protein 8; COX2, cytochrome c oxidase subunit 2; IRF9, IFN regulatory factor 9; RAC1, Ras-related C3 botulinum toxin substrate 1; PTK2, protein kinase domain-containing protein; TP53, cellular tumor antigen p53; IL-6, Interleukin-6; IL-18, Interleukin-18.

To verify the representability and reproducibility of RNA-seq data generated from two individuals in each group at each time point on days 1, 3, and 5 post-challenge, we analyzed eight fish in a group on days 1, 3, and 5 by RT-qPCR analysis. RT-qPCR results were consistent with RNA-seq data showing the square of correlation (R2) as 0.84 (Figure S2). Results showed that immune-related genes, such as IL-1β, TNFα, IFN2, IL-8, IRF9, TP53, and IL-6, were markedly upregulated on day 3 (Figure 5).

## 4. Discussion

This study identified potential pathological mechanisms of the highly virulent IHNV strain in the head kidney of rainbow trout at the transcription level. We have assessed the virulence of the IHNV RtCc0517c strain in rainbow trout and confirmed its high virulence. RNA-seq for the head kidney produced more than 7 Gb data with over 90% of Q30 ratio and over 98% of Q20 ratio, providing abundant and correct data for the analysis. Moreover, RT-qPCR results established the dependability and accuracy of RNA-seq data available for deep research of the complexity of the transcriptome. Furthermore, we identified the highest number of DEGs on day 3, revealing that the molecular mechanisms following IHNV infection on day 3 were more complex.

In this study, GO, KEGG, and PPI network analysis on day 1 identified sugar metabolism-related pathways, such as AMPK signaling and glycolysis/gluconeogenesis, and amino acid metabolism-related pathways in response to the virulent IHNV infection. DEGs in those pathways were mostly upregulated on day 1. These results suggest that the infection of virulent IHNV might interfere with the normal cellular process to obtain more cellular resources for the synthesis of viral proteins as well as modulating metabolic pathways. Some viruses drive metabolic pathways in various aspects, such as providing membrane material for the envelopes of viral particles, genomic replication, and meeting energy costs for packaging [24]. For example, in previous studies, the poliovirus uses or modifies the synthesis of glucose, glutamine, and fatty acids [25,26,27]. HCMV also changed the metabolic pathway using fatty acids, amino acids, and sugars [13,28]. Viruses shape host cell metabolism to obtain supplies for virion production and induce the reorganization of the cellular membrane and biosynthesis machinery, accompanied by changes in lipid metabolism [29]. Most viruses currently examined influence aerobic glycolysis (Warburg effect), fatty acid synthesis, and glutaminolysis [24]. It has been shown that ocular infection with herpes simplex virus-1 (HSV-1) changes blood glucose levels; however, it failed to produce infectious progeny in cells without glucose [30]. Moreover, when glucose usage is pharmacologically limited in vivo in the inflammatory phase, lesions will be diminished. However, glucose usage is also limited in the acute phase of infection when the replicating virus is present in the eye. In that case, infected mice became susceptible to the lethal effects of HSV-1 infection since the virus spread to the brain, causing encephalitis [30]. This result highlights the fundamental relationship between cell metabolism, immune response, and viral pathogenesis.

In this study, the activation of the AMPK signaling pathway induced upregulation of G6PC and facilitated glycolysis and glucogenesis followed by other metabolism-related pathways, such as glycosylate and dicarboxylate metabolism and amino acid and carbon metabolism. Importantly, when activated by energy stress, AMPK restores the cellular energy balance by substituting the catabolic, ATP-generating pathways while switching off anabolic, ATP-consuming pathways [31]. Metabolic stresses activate AMPK by inhibiting mitochondrial ATP production or speeding up ATP consumption [31]. Previous transcriptome analysis in olive flounder showed that VHSV infection activated the immune system and protein synthesis, whereas ATP synthesis and antioxidant system activity were suppressed [12].

Glycolysis intermediates (G6PC, ALDOB, FBP1 (Fructose-Bisphosphatase 1), PCK2, GAPDH and PCK1) in the glycolysis and glucogenesis pathways were upregulated by IHNV infection. G6PC is known to catalyze the hydrolysis of d-glucose 6-phosphate to d-glucose and orthophosphate and is a key functioning enzyme involved in gluconeogenesis and glycogenolysis [32]. GAPDH is an important glycolytic enzyme that catalyzes the conversion of glyceraldehyde-3-phosphate into 1,3-bisphosphoglycerate [33]. Nuclear GAPDH plays a pivotal role in controlling the balance between apoptosis and autophagy [33,34]. Microarray analysis of hepatitis C virus (HCV)-infected humans showed that transcription of several metabolic genes was induced, and that the expression level of PCK significantly influenced the regulation of glucose synthesis [32].

Additionally, this study revealed that glutamic-oxaloacetic transaminase 1 (GOT1) and glutamic-oxaloacetic transaminase 2 (GOT2) were upregulated, while the glutaminase 1 (GLS1), glutaminase 2 (GLS2), glutamate-ammonia ligase (GLUL), and glutamine-fructose-6-phosphate transaminase 2 (GFPT2) genes were downregulated, impacting changes in glutamine metabolism. Glutamine is a non-essential amino acid required for multiple metabolic pathways and can be used as an intermediate in tricarboxylic citric acid (TCA) cycles [35]. The absence of glutamine decreased ATP levels and significantly decreased virus production in HCMV-infected HFF cells [14]. The infected cells’ carbon source usage modifications increase the available energy for virus replication and virion production, thus providing specific cellular substrates for viral particles and creating viral replication niches, subsequently increasing infected cell survival [24].

In KEGG pathway analysis, complement and coagulation cascades were commonly identified at all time points. The activation of the complement system is known to induces the phagocytic cells to recognize and phagocytose foreign pathogens by binding to their surfaces [36]. Additionally, this system contributes to homeostasis as a major regulator of the inflammatory response. However, over-activating the complement system interferes with homeostasis, consequentially damaging immunity balance [36]. Highly virulent IHNV activated the complement pathway at an earlier time point than low virulent IHNV [8]. This study indicated that complement C3 (C3) was upregulated on days 1, 3, and 5, complement factor H (CFH) on day 1, and complement C3a receptor 1 (C3AR1) and complement C7 (C7) on day 3. Conversely, complement factor B (CFB), CFH, and complement C5a receptor 1 (C5AR1) were downregulated on day 3, and MBL-associated serine protease 1 (MASP1) and complement C8 gamma chain (C8G) were downregulated on days 3 and 5. Therefore, it can be postulated that the initial stage of highly virulent IHNV infection stimulates the complement system’s alternative pathway. F5 upregulated the coagulation cascade, alpha-2-macroglobulin (A2M), and the coagulation factor XIII A chain (F13A1) on day 1, and coagulation factor III (F3), coagulation factor V (F5), and serpin family E member 1 (SERPINE1) on day 3, but downregulated overall. The downregulated coagulation cascade results in systemic bleeding due to IHNV infection.

Furthermore, in this study, immune-related GO terms and KEGG pathways were identified on day 3 with the highest centrality of TNF as pattern recognition receptor (PRR) signaling pathways, such as RIG-1-like receptor (RLR), the TLR signaling pathway, NOD-like receptors, and the cytosolic DNA-sensing pathway, and cytokine signaling pathways, such as cytokine–cytokine receptor interaction and the TNF signaling pathway, were identified on day 3. Most immune gene pathways were also upregulated on day 3. DDX58 (RIG-1), IFIH1 (interferon induced with helicase C domain 1), and laboratory of genetics and physiology 2 (LGP2) in the RLR pathway were upregulated on days 3 and 5 in this study. The RLR pathway is a receptor that recognizes viral RNA, with RIG-1, MDA5, and glucagon-like peptide 2 (GLP2) as the principal members [37]. RIG-1 is also an intermediate that detects double-stranded RNA, with LGP2 playing a vital role as a negative regulator [37]. A previous study showed that MDA5 expression inhibited the proliferation of VHSV, hirame rhabdovirus virus, and infectious pancreatic necrosis virus in hirame natural embryo cells [38]. TANK-binding kinase 1 (TBK1), interferon regulatory factor 3 (IRF3), and interferon regulatory factor 7 (IRF7) were also activated, finally leading to the expression of essential cytokines and chemokines in innate immunity [37].

Meanwhile, activation of immune pathways on day 3 was delayed in high virulent IHNV-infected rainbow trout since the immune-related GO terms and KEGG pathways were identified on day 1 in low virulent IHNV-infected fish [8]. IHNV proteins are formed using metabolic pathways and delay various immune responses by interfering with many immune response stages. This delayed induction of immune gene expression plays a critical role in the pathogenesis of virulent IHNV in rainbow trout. Certainly, the negative regulation of type I IFN production-related GO terms was identified at all time points during this study. Notably, rhabdoviruses directly interfere with important immune effector functions, including the IFN system, to avoid immune control [39]. In this study, TLR2 was downregulated on day 3. TLRs are key pattern recognition receptors that recognize the outer viral membrane proteins in the innate immunity of fish [40]. Previously, TLR2 was downregulated in the first stages of a high replication rate of VHSV in the head kidney of olive flounder, indicating virus-induced immunosuppression [41]. IHNV proteins interfere with host signal transduction and gene expression to evade host immune response. Previous studies revealed that N and M proteins of IHNV prevent the response of IFN, L protein initiates NF-kβ, and NV protein restricts NF-kβ [11,42,43]. Therefore, the activation of these genes would play an important role in the immune response and regulate energy metabolism. The virulent IHNV uses numerous metabolic pathways of the host for viral replication and assembly in the first stages of infection.

Interestingly, CAMs and ECM–receptor pathway terms were identified in IHNV-infected fish on day 5, showing the highest centrality of matrix metalloproteinase-9 (MMP9), with downregulated expression. MMP9, one of the widely investigated MMPs, is an important protease that plays a vital role in various biological processes, such as wound healing [44]. Additionally, MMP9 can degrade many ECM proteins through proteolytic cleavage to regulate ECM remodeling [44]. Therefore, the infected fish failed to heal the wound caused by IHNV infection due to the infected fish exhibiting lesions that led to death, although the infected fish induced immune responses against the virus, as indicated in RNA-seq data.

## 5. Conclusions

Summarily, IHNV infection changed the gene expression patterns in vital immune organs of rainbow trout during the first 5 days, activated the energy metabolic pathways, and consumed the energy for viral replication at the early stage of day 1. Additionally, virulent IHNV infection induced the defense mechanism of infected fish by upregulating immune and inflammatory pathways through PRR signaling; however, this was delayed until day 3 or later with the less virulent IHNV infection. It can be postulated that virulent IHNV induces pathogenesis by controlling host metabolism, delaying immune protection, and causing wound healing failure, resulting in the death of fish.

## Figures and Tables

**Table 1:** Comparative analysis of DEGs between groups 1 and 2.

Time (Day)	Group 1	Group 2	Number of Genes		
			Total ^a^	Up ^b^	Down ^c^
1	Control	IHNV	618	525	93
3	Control	IHNV	2626	1236	1390
5	Control	IHNV	774	482	292

^a^ Total number of DEGs in groups 1 and 2. ^b^ Group 2 is more upregulated than group 1. ^c^ Group 2 is more downregulated than group 1.

**Table 2:** GO terms commonly identified at all time points in IHNV-infected fish.

Category	GO ID	GO Terms	Fold Enrichments		
			Day 1	Day 3	Day 5
BP	GO:0032480	Negative regulation of type I IFN production	8.63	5.17	10.87
	GO:0048661	Positive regulation of smooth muscle cell proliferation	5.04	2.95	6.79
	GO:0050718	Positive regulation of IL-1β secretion	9.81	4.03	7.41
	GO:0051607	Defense response to virus	2.35	2.48	3.21
	GO:0002576	Platelet degranulation	3.77	2.58	2.77
	GO:0009615	Response to virus	2.75	2.72	4.45
	GO:0010575	Positive regulation of VEGF production	6.40	3.28	6.04
CC	GO:0031093	Platelet alpha granule lumen	5.71	3.31	3.80
	GO:0016323	Basolateral plasma membrane	3.49	2.27	3.25
	GO:0009925	Basal plasma membrane	7.48	2.65	5.57

IFN, interferon; IL, interleukin; VEGF, vascular endothelial growth factor.

**Table 3:** Top GO terms identified at each point in IHNV-infected fish.

Category	Day 1		Day 3		Day 5	
	GO Terms	F *	GO Terms	F *	GO Terms	F *
BP	Gluconeogenesis	10	Inflammatory response	2	Cell adhesion	3
	Transmembrane transport	3	ECM organization	3	Negative regulation of type I IFN production	11
	Cellular response to hypoxia	4	Cell adhesion	2	ECM organization	4
	Transport	2	Leukocyte migration	3	Positive regulation of smooth muscle cell proliferation	7
	Sodium ion transport	5	Defense response to virus	2	Response to virus	4
	Negative regulation of type I IFN production	9	Negative regulation of type I IFN production	5	Positive regulation of IFNα production	17
	Anion transmembrane transport	9	Cytokine-mediated signaling pathway	3	ERBB2 signaling pathway	8
	Spinal cord development	9	Cellular response to mechanical stimulus	3	Positive regulation of IFNβ production	9
	Response to organic cyclic compound	6	Response to virus	3	Defense response to virus	3
	Cellular response to cAMP	6	TGFβ receptor signaling pathway	3	Negative regulation of endopeptidase activity	4
	Regulation of intracellular pH	7	Positive regulation of ERK1 and ERK2 cascade	2	Innate immune response	2
	Positive regulation of IL-1β secretion	10	Cell–matrix adhesion	3	C21-steroid hormone biosynthetic process	18
	Chloride transport	6	TNF-mediated signaling pathway	3	Glucocorticoid biosynthetic process	18
	Innate immune response	2	Platelet degranulation	3	Response to drug	2
	Positive regulation of smooth muscle cell proliferation	5	Peptidyl-tyrosine phosphorylation	2	Sterol metabolic process	10
CC	Apical plasma membrane	4	ECM	3	ECM	4
	Brush border membrane	7	Cell surface	2	Extracellular space	2
	Basolateral plasma membrane	3	Extracellular vesicle	4	Basement membrane	6
	Platelet alpha granule lumen	6	External side of plasma membrane	2	Cell surface	2
	Stereocilium	8	Proteinaceous ECM	2	Proteinaceous ECM	3
MF	Transporter activity	3	Integrin binding	3	Protein homodimerization activity	2
	Pyridoxal phosphate binding	6	Fibronectin binding	6	NAD+ADP-ribosyltransferase activity	9
	Enzyme binding	2	Collagen binding	4	Integrin binding	4
	Transaminase activity	13	Receptor activity	2	ECM structural constituent	5
	Receptor binding	2	Laminin binding	5	Phosphatidylserine binding	7

The order of the list is itemized from the lowest *p*-value. * F, fold enrichment.

**Table 4:** KEGG pathways commonly identified in IHNV-infected fish.

Category	Pathway ID	Pathway Terms	Fold Enrichments		
			Day 1	Day 3	Day 5
Immune system	hsa04610	Complement and coagulation cascades	3.77	2.47	3.47
Amino acid metabolism	hsa00260	Glycine, serine, and threonine metabolism	5.72	2.19	NI
Excretory system	hsa04964	Proximal tubule bicarbonate reclamation	8.08	2.88	NI
Membrane transport	hsa02010	ABC transporters	4.23	NI	4.67
Infectious disease	hsa05144	Malaria	NI	2.51	4.89
	hsa05162	Measles	NI	2.07	2.57
Signal transduction	hsa04514	Cell adhesion molecules	NI	2.40	3.13

NI, not identified or ignored, as *p*-value > 0.05 or fold enrichments <2.0.

**Table 5:** KEGG pathways uniquely identified at each time point in IHNV-infected fish.

Time	Pathway ID	Pathway Terms	No. of DEGs	F *
Day 1	hsa04976	Bile secretion	10	5.39
	hsa03320	PPAR signaling pathway	9	4.99
	hsa01200	Carbon metabolism	11	3.62
	hsa01130	Biosynthesis of antibiotics	15	2.63
	hsa00340	Histidine metabolism	5	8.45
	hsa01230	Biosynthesis of amino acids	8	4.13
	hsa00630	Glyoxylate and dicarboxylate metabolism	5	6.89
	hsa00330	Arginine and proline metabolism	6	4.46
	hsa04920	Adipocytokine signaling pathway	7	3.72
	hsa04152	AMPK signaling pathway	9	2.72
	hsa04978	Mineral absorption	5	4.23
	hsa00010	Glycolysis/Gluconeogenesis	6	3.33
	hsa00410	b-Alanine metabolism	4	4.8
Day 3	hsa04060	Cytokine-cytokine receptor interaction	52	2.03
	hsa04514	Cell adhesion molecules (CAMs)	36	2.4
	hsa04380	Osteoclast differentiation	34	2.46
	hsa04064	NF-kappa B signaling pathway	23	2.5
	hsa04620	Toll-like receptor signaling pathway	26	2.32
	hsa05160	Hepatitis C	30	2.14
	hsa04668	TNF signaling pathway	24	2.13
	hsa05140	Leishmaniasis	17	2.27
	hsa00250	Alanine, aspartate, and glutamate metabolism	11	2.98
	hsa05144	Malaria	13	2.51
	hsa04622	RIG-I-like receptor signaling pathway	16	2.17
	hsa05410	Hypertrophic cardiomyopathy (HCM)	17	2.07
	hsa04672	Intestinal immune network for IgA production	12	2.42
	hsa04621	NOD-like receptor signaling pathway	13	2.2
	hsa00220	Arginine biosynthesis	7	3.32
	hsa04623	Cytosolic DNA-sensing pathway	14	2.07
	hsa05321	Inflammatory bowel disease (IBD)	14	2.07
	hsa04930	Type II diabetes mellitus	11	2.17
	hsa04964	Proximal tubule bicarbonate reclamation	7	2.88
	hsa04710	Circadian rhythm	8	2.45
	hsa00260	Glycine, serine, and threonine metabolism	9	2.19
Day 5	hsa04913	Ovarian steroidogenesis	9	6.29
	hsa05164	Influenza A	16	3.15
	hsa04514	Cell adhesion molecules (CAMs)	13	3.13
	hsa00140	Steroid hormone biosynthesis	8	4.72
	hsa05144	Malaria	7	4.89
	hsa05168	Herpes simplex infection	13	2.43
	hsa05161	Hepatitis B	10	2.36
	hsa04512	ECM–receptor interaction	7	2.75
	hsa04974	Protein digestion and absorption	7	2.72
	hsa00760	Nicotinate and nicotinamide metabolism	4	4.72
	hsa04622	RIG-I-like receptor signaling pathway	6	2.93
	hsa05140	Leishmaniasis	6	3.17

The order of the list is from the lowest *p*-value (<0.05). * F, fold enrichment.

**Table 6:** The top ten nodes of centrality analysis of the PPI network in the IHNV injected group.

Time (Day)	Category *	Accession ID	Node Name	Closeness Centrality **	Betweenness Centrality ***
1	12, 13, 14	ENSP00000229239	GAPDH	0.637	0.213
	4, 11, 13, 14, 15	ENSP00000319814	PCK1	0.586	0.051
	12, 13, 14, 16	ENSP00000302620	AGXT	0.574	0.074
	11	ENSP00000295834	FABP1	0.563	0.064
	4	ENSP00000312987	HNF4A	0.558	0.053
	12, 13, 14	ENSP00000363988	ALDOB	0.542	0.021
	4, 11, 13, 14, 15	ENSP00000216780	PCK2	0.532	0.016
	4, 11, 14	ENSP00000253801	G6PC	0.523	0.035
	16	ENSP00000291670	FTCD	0.523	0.037
	17	ENSP00000266088	SLC5A1	0.504	0.064
	1, 2, 3, 4, 5, 7, 8	ENSP00000398698	TNF	0.611	0.141
3	1, 2, 3, 4, 5, 7, 8	ENSP00000398698	TNF	0.611	0.141
	1, 2, 3, 4, 5, 7	ENSP00000263341	IL1B	0.558	0.119
	1, 2, 4, 5	ENSP00000451828	AKT1	0.553	0.094
	1, 2, 5, 7	ENSP00000354394	STAT1	0.546	0.054
	1, 2, 7	ENSP00000260010	TLR2	0.543	0.041
	1	ENSP00000275493	EGFR	0.524	0.048
	1, 2, 3, 4	ENSP00000361359	CD40	0.520	0.021
	1, 2, 4, 5, 7	ENSP00000360266	JUN	0.519	0.047
	1, 3, 4	ENSP00000294728	VCAM1	0.512	0.030
	1, 2	ENSP00000370034	TLR7	0.508	0.016
5	1	ENSP00000361405	MMP9	0.636	0.107
	1	ENSP00000360266	JUN	0.631	0.156
	1	ENSP00000263341	IL1B	0.614	0.083
	11	ENSP00000302665	IGF1	0.603	0.101
	1	ENSP00000354394	STAT1	0.565	0.029
	1, 2	ENSP00000245907	C3	0.547	0.050
	11, 18	ENSP00000478561	CYP1B1	0.538	0.056
	11	ENSP00000261693	SCARB1	0.530	0.044
	1	ENSP00000243077	LRP1	0.530	0.010
	1, 3	ENSP00000355751	THBS2	0.530	0.027

* Categories of the PPI network showing a node’s involvement are represented by numbers: 1. Infectious disease, 2. The immune system, 3. Signaling molecules and interaction, 4. Signal transduction, 5. Development and regeneration, 6. Folding, sorting, and degradation, 7. Immune disease, 8. Endocrine and metabolic disease, 9. Cancer: overview, 10. Cellular community, 11. Endocrine system, 12. Global and overview maps, 13. Metabolism of terpenoids and polyketides, 14. Carbohydrate metabolism, 15. Excretory system, 16. Amino acid metabolism, 17. Digestive system, 18. Lipid metabolism. ** Closeness Centrality: Closeness coefficient shows the distance between a node and other nodes in the network. If very short, this indicates that the point is the center of the whole network. The larger the value, the closer the node is to the center of the network. *** Betweenness Centrality: Median Centrality reflects the role of a node in connection with other nodes. The larger the value, the more important the node is in maintaining the close connection of the whole network.

## Data Availability

The data that support the findings of this study are available from the corresponding author upon reasonable request.

## Supplementary Materials

The following supporting information can be downloaded at: https://www.mdpi.com/article/10.3390/v14050859/s1, Figure S1: Visualization of qualities of sequencing raw data. (A) Throughput of total raw data; (B) Total read count of raw data; (C) GC/AT content of raw data; (D) Q20/Q30 scores of raw data, Figure S2: Quality assessment and comparison of transcriptome data quality between control and IHNV groups. (A) Correlation matrix of the transcriptome data of all samples. (B) Summary of the differentially expressed genes in the control and IHNV groups. (C) Correlation analysis of RT-qPCR and RNA-seq. Correlation of fold change analyzed by data obtained using RT-qPCR (*x*-axis) with RNA-seq platform (*y*-axis), Table S1: Primers used in this study, Table S2: Summary statistics for the sequencing data of the 12 samples, Table S3: Statistics analysis of clean reads mapping onto a reference genome, Table S4: Number of GO terms identified in GO enrichment analysis of the IHNV group, Table S5: The top ten GO terms identified in IHNV-infected fish on day 1. The order of the list is itemized from the lowest *p*-value., Table S6: The top ten GO terms identified in IHNV-infected fish on day 3, Table S7: The top ten GO terms identified in IHNV-infected fish on day 5, Table S8: Summary of DEGs in metabolism-related KEGG pathways, Table S9: Summary of DEGs in immune-related KEGG pathways.
