# Supplementary material for: Pathogenic Mechanism of a Highly Virulent Infectious Hematopoietic Necrosis Virus in Head Kidney of Rainbow Trout (Oncorhynchus mykiss) Analyzed by RNA-Seq Transcriptome Profiling
Source: Viruses. 2022 Apr 21;14(5):859. doi: 10.3390/v14050859 (PMC9143916; doi:10.3390/v14050859)

Supplementary Data

Table S1. Primers used in this study.

Table S2. Summary statistics for the sequencing data of the 12 samples.

Table S3. Statistics analysis of clean reads mapping onto a reference genome.

Table S4. Number of GO terms identified in GO enrichment analysis of IHNV group.

Table S5. The top ten GO terms identified in IHNV infected fish at day 1. The order of the list is itemized from the lowest *P*-value.

Table S6. The top ten GO terms identified in IHNV infected fish at day 3. The order of the list is listed from the lowest *P*-value.

Table S7. The top ten GO terms identified in IHNV infected fish at day 5. The order of the list is enumerated from the lowest *P*-value.

Table S8. Summary of DEGs in metabolism-related KEGG pathways.

Table S9. Summary of DEGs in immune-related KEGG pathways.

Fig S1. Visualization of qualities of sequencing raw data. (A) Throughput of total raw data; (B) Total read count of raw data; (C) GC/AT content of raw data; (D) Q20/Q30 scores of raw data

Fig S2. Quality assessment and comparison of transcriptome data quality between control and IHNV groups. A) Correlation matrix of the transcriptome data of all samples. B) Summary of the differentially expressed genes in the control and IHNV groups. (C) Correlation analysis of RT-qPCR and RNA-seq. Correlation of fold change analyzed by data obtained using RT-qPCR (x-axis) with RNA-seq platform (y-axis).

Table S1. Primers used for RT-qPCR and PCR.

Name	Sequences (5’ to 3’)	Objects
IL-18 F<br>IL-18 R	CAATGACATTGCCAAGGCCA<br>GCTTACCAGTGGTTTAGCAG	Q-PCR
IFN2 F<br>IFN2 R	AAAGCTAAAAGCAAAATAAACAGCTCTT<br>TGCAGAGTGTGTGTCATTGCTG	Q-PCR
IL-8 F<br>IL-8 R	AACCGACCGGGTAAAACAAC<br>TGACTTGCTTCACCTGACAG	Q-PCR
IRF9 F<br>IRF9 R	TGAATGCTGACTGGTACTGC<br>AGAGCCTGTCAGTGTTTCTC	Q-PCR
IL-6 F<br>IL-6 R	CGCTCGTGGTGTTAGTTAAG<br>GTGGACGAGCATCTTGATCA	Q-PCR
MT-ATP8 F<br>MT-ATP8 R	TTAGTATTCTCGTGACTGGTTT<br>ATGGTCAGTTTCAGGGTTCG	Q-PCR
TNFα F<br>TNFα R	ACAAACTGTGGACTGAGACC<br>AGGCAGGCCAGAGATGAATA	Q-PCR
IL-1β F<br>IL-1β R	CTTACCACCTTCACCATCCA<br>TCTGTGATCAGGACTGGGTT	Q-PCR
COX2 F<br>COX2 R	TTCCACGACCACGCTCTTAT<br>GACAGTCCAAACGATCTCGA	Q-PCR
TP53 F<br>TP53 R	CTCACCAAGAGAGTTGCCAA<br>GGCTTGCTGAAATGACATGG	Q-PCR
PTK2 F<br>PTK2 R	CATCCCAACACTCTCCACTA<br>GTGGCATGTGACATGTTTGG	Q-PCR
RAC1 F<br>RAC1 R	CAGTGTTTGACGAAGCCATC<br>CAGACTGCAGTGTGACTTC	Q-PCR
EF-1α F<br>EF-1α R	CAGGGAGAAGATGACCCAGA<br>CATAGATGGGCACTGTGTGG	Q-PCR
IHNV G F<br>IHNV G R	TCACCCTGCCAGACTCATTGG<br>ATAGATGGAGCCTTTGTGCAT	viral infection confirmation
IHNV NV F<br>IHNV NV R	AGCATCAAATCCGTACCC<br>GCGAGACTCACAGAGAA	viral infection confirmation

Table S2. Summary statistics for the sequencing data of the 12 samples.

Time (day)	Sample	Total Bases (bp)	Raw reads	Clean reads	GC (%)	AT (%)	Q20 (%)	Q30 (%)
**1**	PBS 1	11,755,507,362	116,391,162	113,451,896 (97.6%)	48.80	51.20	98.44	95.44
	PBS 2	7,623,335,368	75,478,568	73,385,868 (97.4%)	49.58	50.42	98.30	95.12
	IHNV 1	7,953,068,250	78,743,250	76,460,808 (97.3%)	49.38	50.62	98.24	94.99
	IHNV 2	8,387,531,466	83,044,866	80,652,626 (97.3%)	49.14	50.86	98.27	95.08
**3**	PBS 1	7,837,444,662	77,598,462	75,469,696 (97.5%)	49.09	50.91	98.36	95.32
	PBS 2	8,154,824,032	80,740,832	78,619,084 (97.5%)	48.88	51.12	98.38	95.33
	IHNV 1	7,115,593,016	70,451,416	68,576,948 (97.5%)	49.39	50.61	98.39	95.37
	IHNV 2	8,880,701,740	87,927,740	85,558,304 (97.5%)	50.19	49.81	98.33	95.17
**5**	PBS 1	8,916,525,228	88,282,428	86,223,008 (97.8%)	49.47	50.53	98.56	95.78
	PBS 2	9,910,185,650	98,120,650	95,487,188 (97.5%)	49.0	51.0	98.37	95.30
	IHNV 1	9,518,450,282	94,242,082	91,667,032 (97.4%)	48.66	51.34	98.36	95.36
	IHNV 2	9,466,931,798	93,731,998	91,080,060 (97.3%)	48.99	51.01	98.28	95.13

Table S3. Statistics analysis of clean reads mapping onto a reference genome.

Time (day)	Sample	No. of mapped reads	Percentages of mapped reads (%)	No. of uniquely mapped reads	Percentages of uniquely mapped reads (%)
**1**	PBS 1	98,986,537	87.2	90,744,204	80.0
	PBS 2	64,278,162	87.6	59,266,630	80.8
	IHNV 1	66,356,695	86.8	58,031,227	75.9
	IHNV 2	70,157,774	87.0	62,518,161	77.5
**3**	PBS 1	66,021,957	87.5	60,649,197	80.4
	PBS 2	68,139,289	86.7	62,864,439	80.0
	IHNV 1	59,659,308	87.0	52,541,290	76.6
	IHNV 2	75,339,430	88.1	64,858,921	75.8
**5**	PBS 1	75,523,041	87.6	69,638,263	80.8
	PBS 2	83,154,279	87.1	76,600,416	80.2
	IHNV 1	77,318,053	84.3	69,731,060	76.1
	IHNV 2	77,667,781	85.3	70,465,199	77.4

Table S4. Number of GO terms identified in GO enrichment analysis of IHNV group.

Time (day)	Comparison group (Con vs.)	Total	GO categories		
			BP	CC	MF
**1**	IHNV	115	67	18	30
**3**	IHNV	341	244	37	60
**5**	IHNV	118	75	27	16

Table S5. The top ten GO terms identified in IHNV infected fish at day 1. The order of the list is itemized from the lowest *P*-value.

**Category**	**GO ID**	**GO terms**	**No. of DEGs**	**P-value**
Biological process	GO:0006094	gluconeogenesis	10	0.0000
	GO:0055085	transmembrane transport	18	0.0001
	GO:0071456	cellular response to hypoxia	10	0.0004
	GO:0006810	transport	20	0.0005
	GO:0006814	sodium ion transport	9	0.0006
	GO:0032480	negative regulation of type I IFN production	6	0.0006
	GO:0098656	anion transmembrane transport	6	0.0006
	GO:0021510	spinal cord development	6	0.0006
	GO:0014070	response to organic cyclic compound	7	0.0009
	GO:0071320	cellular response to cAMP	7	0.0012
Cellular Component	GO:0070062	extracellular exosome	108	0.0000
	GO:0016324	apical plasma membrane	26	0.0000
	GO:0005886	plasma membrane	126	0.0001
	GO:0031526	brush border membrane	8	0.0001
	GO:0005829	cytosol	104	0.0002
	GO:0016323	basolateral plasma membrane	14	0.0002
	GO:0005887	integral component of plasma membrane	53	0.0002
	GO:0005783	endoplasmic reticulum	34	0.0009
	GO:0031093	platelet alpha granule lumen	7	0.0013
	GO:0032420	stereocilium	5	0.0028
Molecular function	GO:0005215	transporter activity	15	0.0002
	GO:0030170	pyridoxal phosphate binding	8	0.0003
	GO:0019899	enzyme binding	19	0.0007
	GO:0008483	transaminase activity	4	0.0029
	GO:0005102	receptor binding	18	0.0033
	GO:0003824	catalytic activity	12	0.0041
	GO:0008656	cysteine-type endopeptidase activator activity involved in apoptotic process	4	0.0064
	GO:0004879	RNA polymerase II transcription factor activity, ligand-activated sequence-specific DNA binding	5	0.0089
	GO:0008237	metallopeptidase activity	7	0.0106
	GO:0008201	heparin binding	10	0.0114

Table S6. The top ten GO terms identified in IHNV infected fish at day 3. The order of the list is listed from the lowest *P*-value.

Category	GO ID	GO terms	No. of DEGs	P-value
Biological process	GO:0006954	inflammatory response	84	0.0000
	GO:0030198	extracellular matrix organization	50	0.0000
	GO:0007165	signal transduction	168	0.0000
	GO:0007155	cell adhesion	83	0.0000
	GO:0050900	leukocyte migration	33	0.0000
	GO:0051607	defense response to virus	37	0.0000
	GO:0032480	negative regulation of type I IFN production	14	0.0000
	GO:0019221	cytokine-mediated signaling pathway	31	0.0000
	GO:0006955	immune response	69	0.0000
	GO:0071260	cellular response to mechanical stimulus	21	0.0000
Cellular Component	GO:0005887	integral component of plasma membrane	205	0.0000
	GO:0005886	plasma membrane	482	0.0000
	GO:0031012	extracellular matrix	67	0.0000
	GO:0009986	cell surface	100	0.0000
	GO:0005829	cytosol	394	0.0000
	GO:0070062	extracellular exosome	341	0.0000
	GO:0005615	extracellular space	185	0.0000
	GO:0005737	cytoplasm	552	0.0000
	GO:1903561	extracellular vesicle	18	0.0000
	GO:0009897	external side of plasma membrane	42	0.0000
Molecular function	GO:0005178	integrin binding	31	0.0000
	GO:0005515	protein binding	880	0.0000
	GO:0001968	fibronectin binding	13	0.0000
	GO:0005518	collagen binding	19	0.0000
	GO:0004872	receptor activity	42	0.0000
	GO:0043236	laminin binding	11	0.0000
	GO:0042802	identical protein binding	101	0.0000
	GO:0019899	enzyme binding	53	0.0001
	GO:0001618	virus receptor activity	18	0.0001
	GO:0050431	transforming growth factor beta binding	8	0.0003

Table S7. The top ten GO terms identified in IHNV infected fish at day 5. The order of the list is enumerated from the lowest *P*-value.

Category	GO ID	GO terms	No. of DEGs	P-value
Biological process	GO:0007155	cell adhesion	30	0.0000
	GO:0032480	negative regulation of type I IFN production	8	0.0000
	GO:0030198	extracellular matrix organization	18	0.0000
	GO:0048661	positive regulation of smooth muscle cell proliferation	10	0.0000
	GO:0009615	response to virus	12	0.0001
	GO:0032727	positive regulation of interferon-alpha production	5	0.0001
	GO:0038128	ERBB2 signaling pathway	7	0.0003
	GO:0032728	positive regulation of interferon-beta production	6	0.0004
	GO:0051607	defense response to virus	13	0.0008
	GO:0010951	negative regulation of endopeptidase activity	11	0.0008
Cellular Component	GO:0070062	extracellular exosome	122	0.0000
	GO:0031012	extracellular matrix	30	0.0000
	GO:0005615	extracellular space	65	0.0000
	GO:0005604	basement membrane	11	0.0000
	GO:0005576	extracellular region	65	0.0000
	GO:0005887	integral component of plasma membrane	59	0.0000
	GO:0009986	cell surface	30	0.0000
	GO:0005578	proteinaceous extracellular matrix	19	0.0001
	GO:0005886	plasma membrane	133	0.0001
	GO:0030424	axon	16	0.0003
Molecular function	GO:0042803	protein homodimerization activity	37	0.0001
	GO:0003950	NAD+ ADP-ribosyltransferase activity	6	0.0006
	GO:0042802	identical protein binding	34	0.0010
	GO:0005178	integrin binding	10	0.0012
	GO:0005201	extracellular matrix structural constituent	8	0.0013
	GO:0001786	phosphatidylserine binding	6	0.0021
	GO:0003727	single-stranded RNA binding	6	0.0040
	GO:0042626	ATPase activity, coupled to transmembrane movement of substances	6	0.0045
	GO:0008201	heparin binding	11	0.0066
	GO:0019966	interleukin-1 binding	3	0.0086

Table S8. Summary of DEGs in metabolism-related KEGG pathways.

Gene name	Full name	Day 1		Day 3		Day 5	
		Fold change	P-value	Fold change	P-value	Fold change	P-value
**AMPK signaling pathway**							
CPT1A	Carnitine palmitoyltransferase 1A	5.22	0.0222				
FBP1	Fructose-bisphosphatase 1	5.21	0.0011				
G6PC	Glucose-6-phosphatase catalytic subunit 1	4.54	0.0112				
HNF4A	Hepatocyte nuclear factor 4 alpha	4.61	0.0030				
MTOR	Mechanistic target of rapamycin kinase	-2.92	0.0082				
PCK1	Phosphoenolpyruvate carboxykinase 1	4.05	0.0140				
PCK2	Phosphoenolpyruvate carboxykinase 2, mitochondrial	4.3	0.0009				
PPARGC1A	PPARG coactivator 1 alpha	2.4	0.0285				
IGF1R	Insulin like growth factor 1 receptor	2.18	0.0077				
**Alanine, aspartate and glutamate metabolism**							
ABAT	4-aminobutyrate aminotransferase			-2.76	0.0328		
AGXT	Alanine--glyoxylate and serine--pyruvate aminotransferase			-6.4	0.0126		
AGXT2	Alanine--glyoxylate aminotransferase 2			-4.44	0.0114		
ALDH4A1	Aldehyde dehydrogenase 4 family member A1			3.92	0.0001		
ASL	Argininosuccinate lyase			-2.38	0.0001		
GFPT2	Glutamine-fructose-6-phosphate transaminase 2			-2.45	0.0067		
GLS	Glutaminase			-3.32	0.0052		
GLUL	Glutamate-ammonia ligase			-3.13	0.0001		
GOT2	Glutamic-oxaloacetic transaminase 2			2.11	0.0001		
NIT2	Nitrilase family member 2			-2.34	0.0001		
GLS2	Glutaminase 2			-2.51	0.0201		
**Arginine biosynthesis**							
ARG1	Arginase 1			2.88	0.0001		
ARG2	Arginase 2			2.58	0.0088		
ASL	Argininosuccinate Lyase			-2.38	0.0001		
GLS	Glutaminase			-2.38	0.0001		
GLUL	Glutamate-Ammonia Ligase			-3.13	0.0001		
GOT2	Glutamic-Oxaloacetic Transaminase 2			2.11	0.0001		
GLS2	Glutaminase 2			-2.51	0.0201		
**Arginine and proline metabolism**							
ARG2	Arginase 2	-2.81	0.0291				
CARNS1	Carnosine synthase 1	2.23	0.0022				
CNDP1	Carnosine dipeptidase 1	3.32	0.0001				
CNDP2	Carnosine dipeptidase 2	3.15	0.0004				
GAMT	Guanidinoacetate N-methyltransferase	4.23	0.0001				
GOT1	Glutamic-oxaloacetic transaminase 1	2.91	0.0013				
**β-Alanine metabolism**							
ALDH6A1	Aldehyde dehydrogenase 6 family member A1	4.4	0.0001				
CNDP2	Carnosine dipeptidase 2	3.15	0.0004				
CNDP1	Carnosine dipeptidase 1	3.32	0.0001				
CARNS1	Carnosine synthase 1	2.23	0.0022				
**Biosynthesis of amino acids**							
ALDOB	Aldolase, fructose-bisphosphate B	3.09	0.0001				
ARG2	Arginase 2	-2.81	0.0291				
GAPDH	Glyceraldehyde-3-phosphate dehydrogenase	2.32	0.0001				
GOT1	Glutamic-oxaloacetic transaminase 1	2.91	0.0013				
GPT2	Glutamic--pyruvic transaminase 2	7.78	0.0107				
MAT1A	Methionine adenosyltransferase 1A	2.1	0.0011				
SDS	Serine dehydratase	3.01	0.0009				
BCAT1	Branched chain amino acid transaminase 1	8.01	0.0056				
**Biosynthesis of antibiotics**							
AGXT	Alanine--glyoxylate and serine--pyruvate aminotransferase	5.58	0.0001				
ALDOB	Aldolase, fructose-bisphosphate B	3.09	0.0001				
ARG2	Arginase 2	-2.81	0.0291				

BCAT1	Branched chain amino acid transaminase 1	8.01	0.0056				
FBP1	Fructose-bisphosphatase 1	5.21	0.0011				
GAPDH	Glyceraldehyde-3-phosphate dehydrogenase	2.32	0.0001				
GCSH	Glycine cleavage system protein H	4.46	0.0165				
GLDC	Glycine decarboxylase	2.01	0.0020				
GOT1	Glutamic-oxaloacetic transaminase 1	2.91	0.0013				
HAO1	Hydroxyacid oxidase 1	5.28	0.0283				
HAO2	Hydroxyacid oxidase 2	3.42	0.0067				
PAPSS2	3'-Phosphoadenosine 5'-phosphosulfate synthase 2	3.9	0.0001				
PCK1	Phosphoenolpyruvate carboxykinase 1	4.05	0.0140				
PCK2	Phosphoenolpyruvate carboxykinase 2, mitochondrial	4.3	0.0009				
SDS	Serine dehydratase	3.01	0.0009				
**Carbon metabolism**							
AGXT	Alanine--glyoxylate and serine--pyruvate aminotransferase	5.58	0.0001				
ALDH6A1	Aldehyde dehydrogenase 6 family member A1	4.4	0.0001				
ALDOB	Aldolase, fructose-bisphosphate B	3.09	0.0001				
FBP1	Fructose-bisphosphatase 1	5.21	0.0011				
GAPDH	Glyceraldehyde-3-phosphate dehydrogenase	2.32	0.0001				
GLDC	Glycine decarboxylase	2.01	0.0020				
GOT1	Glutamic-oxaloacetic transaminase 1	2.91	0.0013				
GPT2	Glutamic--pyruvic transaminase 2	7.78	0.0107				
HAO1	Hydroxyacid oxidase 1	5.28	0.0283				
HAO2	Hydroxyacid oxidase 2	3.42	0.0067				
SDS	Serine dehydratase	3.01	0.0009				
**Glycine, serine and threonine metabolism**							
AGXT2	Alanine--glyoxylate aminotransferase 2			-4.44	0.0114		
AOC3	Amine oxidase copper containing 3			-3.28	0.0001		
GRHPR	Glyoxylate and hydroxypyruvate reductase			-3	0.0458		
PHGDH	Phosphoglycerate dehydrogenase			2.48	0.0001		
AGXT	Alanine--glyoxylate and serine--pyruvate aminotransferase	5.58	0.0001	-6.4	0.0126		
AGXT2	Alanine--glyoxylate aminotransferase 2	2.5	0.0049	-4.44	0.0114		
ALAS1	5'-Aminolevulinate synthase 1			2.81	0.0001		
GAMT	Guanidinoacetate n-methyltransferase	4.23	0.0001	-3.31	0.0001		
GCSH	Glycine cleavage system protein H	4.46	0.0165				
GLDC	Glycine decarboxylase	2.01	0.0020	-4.37	0.0342		
SDS	Serine dehydratase	3.01	0.0009	10.5	0.0001		
**Glycolysis / Gluconeogenesis**							
ALDOB	Aldolase, fructose-bisphosphate B	3.09	0.0001				
FBP1	Fructose-bisphosphatase 1	5.21	0.0011				
GAPDH	Glyceraldehyde-3-phosphate dehydrogenase	2.32	0.0001				
PCK1	Phosphoenolpyruvate carboxykinase 1	4.05	0.0140				
PCK2	Phosphoenolpyruvate carboxykinase 2, mitochondrial	4.3	0.0009				
G6PC	Glucose-6-phosphatase catalytic subunit 1	4.54	0.0112				
**Glyoxylate and dicarboxylate metabolism**							
AGXT	Alanine--glyoxylate and serine--pyruvate aminotransferase	5.58	0.0001				
GCSH	Glycine cleavage system protein H	4.46	0.0165				
GLDC	Glycine decarboxylase	2.01	0.0020				
HAO2	Hydroxyacid oxidase 2	3.42	0.0067				
HAO1	Hydroxyacid oxidase 1	5.28	0.0283				
**Histidine metabolism**							
CARNS1	Carnosine synthase 1	2.23	0.0022				
CNDP1	Carnosine dipeptidase 1	3.32	0.0001				
CNDP2	Carnosine dipeptidase 2	3.15	0.0004				
FTCD	Formimidoyltransferase cyclodeaminase	3.41	0.0001				
HAL	Histidine ammonia-lyase	5.84	0.0001				
**Steroid hormone biosynthesis**							
CYP11A1	Cytochrome P450 family 11 subfamily A member 1					-2.94	0.0001

CYP17A1	Cytochrome P450 family 17 subfamily A member 1	-5.28	0.0001
CYP1B1	Cytochrome P450 family 1 subfamily B member 1	-2.18	0.0407
CYP21A2	Cytochrome P450 family 21 subfamily A member 2	-3.93	0.0001
HSD11B2	Hydroxysteroid 11-beta dehydrogenase 2	-2.95	0.0001
HSD17B3	Hydroxysteroid 17-beta dehydrogenase 3	-2.73	0.0001
UGT2A1	UDP glucuronosyltransferase family 2 member A1 complex locus	-2.39	0.0001
UGT1A6	UDP glucuronosyltransferase family 1 member A6	-2.27	0.0130

Table S9. Summary of DEGs in immune-related KEGG pathways.

Gene name	Full name	Day 1		Day 3		Day 5	
		Fold change	P-value	Fold change	P-value	Fold change	P-value
**Cytokine-cytokine receptor interaction**							
ACVR1	Activin A receptor type 1			-2.05	0.0004		
ACVR1B	Activin A receptor type 1B			2.22	0.0001		
CCL13	C-C motif chemokine ligand 13			-2.7	0.0001		
CCL19	C-C motif chemokine ligand 19			6.73	0.0001		
CCL25	C-C motif chemokine ligand 25			-2.62	0.0001		
CCL4	C-C motif chemokine ligand 4			2.62	0.0001		
CCL4L2	C-C motif chemokine ligand 4 like 2			-2.31	0.0001		
CCR3	C-C motif chemokine receptor 3			-2.45	0.0001		
CCR4	C-C motif chemokine receptor 4			-3.06	0.0011		
CCR5	C-C motif chemokine receptor 5			-3.38	0.0001		
CCR7	C-C motif chemokine receptor 7			-2.75	0.0007		
CCR9	C-C motif chemokine receptor 9			5.09	0.0249		
CD40	CD40 molecule			2.38	0.0001		
CSF2RB	Colony stimulating factor 2 receptor subunit beta			3.08	0.0001		
CXCL11	C-X-C motif chemokine ligand 11			4.73	0.0001		
CXCL12	C-X-C motif chemokine ligand 12			-2.85	0.0001		
CXCR1	C-X-C motif chemokine receptor 1			2.74	0.0001		
CXCR3	C-X-C motif chemokine receptor 3			4.15	0.0001		
CXCR4	C-X-C motif chemokine receptor 4			2.09	0.0007		
FASLG	Fas ligand			3.19	0.0013		
IFNA13	Interferon alpha 13			6.39	0.0041		
IFNA4	Interferon alpha 4			5.99	0.0374		
IL10RA	Interleukin 10 receptor subunit alpha			2.45	0.0001		
IL10RB	Interleukin 10 receptor subunit beta			3.31	0.0001		
IL12B	Interleukin 12B			2.72	0.0006		
IL12RB2	Interleukin 12 receptor subunit beta 2			3.94	0.0026		
IL17RA	Interleukin 17 receptor A			-2.26	0.0442		
IL1B	Interleukin 1 beta			6.36	0.0001		
IL1R1	Interleukin 1 receptor type 1			-2.16	0.0001		
IL1R2	Interleukin 1 receptor type 2			2.81	0.0001		
IL20RA	Interleukin 20 receptor subunit alpha			2.78	0.0001		
IL21R	Interleukin 21 receptor			2.31	0.0001		
IL2RG	Interleukin 2 receptor subunit gamma			2.28	0.0001		
IL7R	Interleukin 7 receptor			-2.32	0.0001		
LIFR	LIF receptor subunit alpha			2.22	0.0001		
NGFR	Nerve growth factor receptor			-3.62	0.0002		
PPBP	Pro-platelet basic protein			6.15	0.0001		
TGFB1	Transforming growth factor beta 1			2.02	0.0001		
TGFB3	Transforming growth factor beta 3			2.92	0.0316		
TGFBR1	Transforming growth factor beta receptor 1			-2.35	0.0267		
TGFBR2	Transforming growth factor beta receptor 2			-2.87	0.0001		
TNF	Tumor necrosis factor			3.21	0.0020		
TNFRSF11A	TNF receptor superfamily member 11a			-4.06	0.0001		
TNFRSF13B	TNF receptor superfamily member 13B			-2.57	0.0003		
TNFRSF1B	TNF receptor superfamily member 1B			3.44	0.0118		
TNFRSF6B	TNF receptor superfamily member 6b			7.8	0.0001		
TNFRSF9	TNF receptor superfamily member 9			3.26	0.0001		
TNFSF12	TNF superfamily member 12			-4.5	0.0001		
TNFSF13	TNF superfamily member 13			3.47	0.0310		
TNFSF14	TNF superfamily member 14			4.12	0.0002		
XCR1	X-C Motif Chemokine Receptor 1			2.75	0.0001		
IL6ST	Interleukin 6 cytokine family signal transducer			4.67	0.0001		
**NF-kappa B signaling pathway**							
CXCL12	C-X-C motif chemokine ligand 12			-2.85	0.0001		
BCL2	BCL2 apoptosis regulator			-2.28	0.0011		
CCL13	C-C motif chemokine ligand 13			-2.7	0.0001		
CCL19	C-C motif chemokine ligand 19			6.73	0.0001		
CCL4	C-C motif chemokine ligand 4			2.62	0.0001		

CCL4L2	C-C motif chemokine ligand 4 Like 2	-2.31	0.0001
CD40	CD40 molecule	2.38	0.0001
CFLAR	CASP8 and FADD like apoptosis regulator	2.85	0.0001
DDX58	DExD/H-box helicase 58	5.14	0.0001
IL1B	Interleukin 1 beta	6.36	0.0001
IL1R1	Interleukin 1 receptor type 1	-2.16	0.0001
NFKBIA	NFKB inhibitor alpha	3.12	0.0001
PTGS2	Prostaglandin-endoperoxide synthase 2	5.95	0.0024
RELB	RELB proto-oncogene, NF-KB subunit	2.04	0.0001
TNF	Tumor necrosis factor	4.69	0.0001
TNFAIP3	TNF alpha induced protein 3	2.15	0.0001
TNFRSF11A	TNF receptor superfamily member 11a	-4.06	0.0001
TNFSF14	TNF superfamily member 14	4.12	0.0002
TRAF2	TNF receptor associated factor 2	3.09	0.0001
TRAF3	TNF receptor associated factor 3	3.43	0.0001
TRIM25	Tripartite motif containing 25	2.64	0.0001
VCAM1	Vascular cell adhesion molecule 1	2.74	0.0001
TRAF1	TNF receptor associated factor 1	3.56	0.0001
**TNF signaling pathway**			
AKT1	Akt serine/threonine kinase 1	2.48	0.0001
BCL3	Bcl3 transcription coactivator	5.31	0.0001
CFLAR	Casp8 and fadd like apoptosis regulator	2.85	0.0001
CREB1	Camp responsive element binding protein 1	-2.25	0.0003
CREB5	Camp responsive element binding protein 5	-2.33	0.0291
IL1B	Interleukin 1 beta	6.36	0.0001
JUN	Jun proto-oncogene, ap-1 transcription factor subunit	3.2	0.0001
MAP2K6	Mitogen-activated protein kinase kinase 6	-4.53	0.0001
MAPK12	Mitogen-activated protein kinase 12	2.11	0.0057
MLKL	Mixed lineage kinase domain like pseudokinase	4.04	0.0001
MMP14	Matrix metallopeptidase 14	2.94	0.0001
NFKBIA	NFkb inhibitor alpha	3.12	0.0001
NOD2	Nucleotide binding oligomerization domain containing 2	2.25	0.0054
PGAM5	Pgam family member 5, mitochondrial serine/threonine protein phosphatase	2.14	0.0001
PIK3R2	Phosphoinositide-3-kinase regulatory subunit 2	-2.2	0.0012
PTGS2	Prostaglandin-endoperoxide synthase 2	5.95	0.0024
SOCS3	Suppressor of cytokine signaling 3	4.9	0.0001
TNF	Tumor necrosis factor	4.69	0.0001
TNFAIP3	TNF alpha induced protein 3	2.15	0.0001
TNFRSF1B	TNF receptor superfamily member 1b	3.44	0.0118
TRAF2	TNF receptor associated factor 2	3.09	0.0001
TRAF3	TNF receptor associated factor 3	3.43	0.0001
VCAM1	Vascular cell adhesion molecule 1	2.74	0.0001
TRAF1	TNF receptor associated factor 1	3.56	0.0001
**NOD-like receptor signaling pathway**			
CASP1	Caspase 1	-3.71	0.0054
IL1B	Interleukin 1 beta	6.36	0.0001
MAPK12	Mitogen-activated protein kinase 12	2.11	0.0057
MEFV	Mefv innate immuity regulator, pyrin	6.76	0.0001
NFKBIA	NFkb inhibitor alpha	3.12	0.0001
NLRP1	NLR family pyrin domain containing 1	2.07	0.0159
NOD2	Nucleotide binding oligomerization domain containing 2	2.25	0.0054
PYCARD	PYD and card domain containing	2.75	0.0001
RIPK2	Receptor interacting serine/threonine kinase 2	3.54	0.0001
SUGT1	SGT1 homolog, mis12 kinetochore complex assembly cochaperone	2.23	0.0001
TNFAIP3	TNF alpha induced protein 3	2.15	0.0001
HSP90AA1	Heat shock protein 90 alpha family class a member 1	4.76	0.0001
TNF	Tumor necrosis factor	4.69	0.0001

**RIG-I-like receptor signaling pathway**			
CYLD	Cyld lysine 63 deubiquitinase	2.12	0.0001
DDX58	Dexd/h-box helicase 58	5.14	0.0001
DHX58	Dexh-box helicase 58	5.59	0.0001
IFIH1	Interferon induced with helicase C domain 1	3.36	0.0001
IFNA13	Interferon alpha 13	6.39	0.0041
IFNA4	Interferon alpha 4	5.99	0.0374
IKBKE	Inhibitor of nuclear factor kappa b kinase subunit epsilon	2.89	0.0001
IL12B	Interleukin 12b	2.72	0.0006
IRF3	Interferon regulatory factor 3	3.39	0.0001
IRF7	Interferon regulatory factor 7	4.2	0.0001
MAPK12	Mitogen-activated protein kinase 12	2.11	0.0057
NFKBIA	Nfkb inhibitor alpha	3.12	0.0001
TNF	Tumor necrosis factor	4.69	0.0001
TRAF3	TNF receptor associated factor 3	3.43	0.0001
TRIM25	Tripartite motif containing 25	2.64	0.0001
TRAF2	TNF receptor associated factor 2	3.09	0.0001
**Toll-like receptor signaling pathway**			
AKT1	Akt serine/threonine kinase 1	2.48	0.0001
CCL4	C-C motif chemokine ligand 4	2.62	0.0001
CCL4L2	C-C motif chemokine ligand 4 like 2	-2.31	0.0001
CD40	CD40 molecule	2.38	0.0001
CTSK	Cathepsin K	-2.46	0.0001
CXCL11	C=X=C motif chemokine ligand 11	2.91	0.0001
IFNA13	Interferon alpha 13	6.39	0.0041
IFNA4	Interferon alpha 4	5.99	0.0374
IKBKE	Inhibitor of nuclear factor kappa b kinase subunit epsilon	2.89	0.0001
IL12B	Interleukin 12b	2.72	0.0006
IL1B	Interleukin 1 beta	6.36	0.0001
IRF3	Interferon regulatory factor 3	3.39	0.0001
IRF5	Interferon regulatory factor 5	2.44	0.0001
IRF7	Interferon regulatory factor 7	4.2	0.0001
JUN	Jun proto-oncogene, ap-1 transcription factor subunit	3.2	0.0001
MAP2K6	Mitogen-activated protein kinase kinase 6	-4.53	0.0001
MAPK12	Mitogen-activated protein kinase 12	2.11	0.0057
NFKBIA	NFkb inhibitor alpha	3.12	0.0001
PIK3R2	Phosphoinositide-3-kinase regulatory subunit 2	-2.2	0.0012
RAC1	RAC family small gtpase 1	2.02	0.0001
STAT1	Signal transducer and activator of transcription 1	2.64	0.0001
TLR2	Toll like receptor 2	-2.47	0.0001
TLR7	Toll like receptor 7	2.14	0.0001
TLR8	Toll like receptor 8	2.54	0.0001
TRAF3	TNF receptor associated factor 3	3.43	0.0001
TNF	Tumor necrosis factor	4.69	0.0001
**Cytosolic DNA-sensing pathway**			
ADAR	Adenosine deaminase rna specific	2.52	0.0001
CASP1	Caspase 1	-3.71	0.0054
CCL4	C-C motif chemokine ligand 4	2.62	0.0001
CCL4L2	C-C motif chemokine ligand 4 like 2	-2.31	0.0001
DDX58	Dexd/h-box helicase 58	5.14	0.0001
IFNA13	Interferon alpha 13	6.39	0.0041
IFNA4	Interferon alpha 4	5.99	0.0374
IKBKE	Inhibitor of nuclear factor kappa b kinase subunit epsilon	2.89	0.0001
IL1B	Interleukin 1 beta	6.36	0.0001
IRF3	Interferon regulatory factor 3	3.39	0.0001
IRF7	Interferon regulatory factor 7	4.2	0.0001
MB21D1 (CGAS)	Cyclic gmp-amp synthase	5.66	0.0001
NFKBIA	NFkb inhibitor alpha	3.12	0.0001

PYCARD	PYD and card domain containing			2.75	0.0001		
**Cell adhesion molecules (CAMs)**							
CADM1	Cell adhesion molecule 1			-2.47	0.0012	-2.61	0.0001
CD22	CD22 molecule			2.7	0.0001		
CD274	CD274 molecule			4.82	0.0001		
CD276	CD276 molecule			3.84	0.0001		
CD28	CD28 molecule			-3.16	0.0001		
CD40	CD40 molecule			2.38	0.0001		
CD58	CD58 molecule			3.57	0.0015	2.46	0.0025
CD58	CD58 molecule			3.57	0.0015		
CLDN1	Claudin 1			5.19	0.0001	2.95	0.0001
CLDN1	Claudin 1			5.19	0.0001		
CLDN19	Claudin 19			2.26	0.0027		
CLDN3	Claudin 3					3.11	0.0483
CLDN4	Claudin 4			-2.5	0.0039		
CLDN5	Claudin 5			2.36	0.0003		
CLDN6	Claudin 6			-3.74	0.0004		
CNTN1	Contactin 1					-3.25	0.016
CNTNAP2	Contactin associated protein 2			-2.52	0.0001		
F11R	F11 receptor			-2.13	0.0003		
ICOSLG	Inducible t cell costimulator ligand			-2.36	0.0001		
ITGAL	Integrin subunit alpha l			2.88	0.0001	2.3	0.0001
ITGA6	Integrin subunit alpha 6			-2.49	0.0023	-2.01	0.0001
ITGA8	Integrin subunit alpha 8			-3.27	0.0485		
ITGA9	Integrin subunit alpha 9			2.32	0.0001	-2.44	0.0485
ITGB1	Integrin subunit beta 1			-2.09	0.0001		
ITGB7	Integrin subunit beta 7			-2.27	0.0001		
JAM2	Junctional adhesion molecule 2			-2.35	0.0070		
JAM3	Junctional adhesion molecule 3			-2.27	0.0001		
MAG	Myelin associated glycoprotein			2.36	0.0001	2.22	0.0001
MPZ	Myelin protein zero			-3.14	0.0002		
MPZ	Myelin protein zero			-3.14	0.0002	-2.39	0.0001
NCAM1	Neural cell adhesion molecule 1			-4.25	0.0001		
NCAM2	Neural cell adhesion molecule 2			-3.27	0.0001		
NFASC	Neurofascin			-2.78	0.0001		
NTNG2	Netrin g2			2.31	0.0001		
OCLN	Occludin			-3.09	0.0082		
PECAM1	Platelet and endothelial cell adhesion molecule 1			-3.61	0.0001		
SDC2	Syndecan 2			3.51	0.0001	2.12	0.0001
SDC4	Syndecan 4			4.27	0.0001		
SIGLEC1	Sialic acid binding ig like lectin 1			2.67	0.0001	2.92	0.0001
VCAM1	Vascular cell adhesion molecule 1			2.74	0.0001		
VCAN	Versican			3.13	0.0001		
HLA-DQB1	Major histocompatibility complex, class ii, dq beta 1					-2.08	0.0015
ICOSLG	Inducible t cell costimulator ligand			-2.36	0.0001		
ITGAL	Integrin subunit alpha l			2.88	0.0001	2.3	0.0001
**Complement and coagulation cascades**							
A2M	Alpha-2-macroglobulin	3.53	0.04305			-2.35	0.0001
C3	Complement c3	3.87	5.00E-05	2.23	0.0001	2.64	0.0001
C3AR1	Complement c3a receptor 1			3.27	0.0001		
C5AR1	Complement c5a receptor 1			-3.14	0.0006		
C7	Complement c7			2.53	0.0001		
C8G	Complement c8 gamma chain			-3.21	0.0008	-3.82	0.0012
CFH	Complement factor h	2.73	0.0011	-2.87	0.0051		
F10	Coagulation factor x			-2.52	0.0008		
F13A1	oagulation factor xiii a chain	2.5	0.02525			-3.56	0.0269
F2R	Coagulation factor ii thrombin receptor			-2.54	0.0490		
F3	Coagulation factor iii, tissue factor			6.46	0.0001	-3.01	0.0348
F5	Coagulation factor v	4.93	0.0013	2.2	0.0391		
F7	Coagulation factor vii					-2.57	0.0148
FGB	Fibrinogen beta chain	-3.85	0.01085	-2.18	0.0076		

FGG	Fibrinogen gamma chain			-2.14	0.0010		
PROS1	Protein S	-2.75	0.0014	-2.49	0.0060		
SERPINE1	Serpin family e member 1			2.21	0.0001		
THBD	Thrombomodulin			-2.82	0.0001		
MASP1	MBL associated serine protease 1			-2.81	0.0214	-2.92	0.0057
PLAT	Plasminogen activator, tissue type			-2.02	0.0001		
**ECM-receptor interaction**							
COL6A6	Collagen type vi alpha 6 chain					2.57	0.0003
HSPG2	Heparan sulfate proteoglycan 2					-2.06	0.0168
ITGA6	Integrin subunit alpha 6			-2.49	0.0023	-2.01	0.0001
THBS2	Thrombospondin 2			7.01	0.0003	2.71	0.0003
TNN	Tenascin N			6	0.0001	2.37	0.0027
TNR	Tenascin R					2.09	0.0058
ITGA9	Integrin subunit alpha 9			2.32	0.0001	-2.44	0.0485
**Intestinal immune network for IgA production**							
CCL25	C-C motif chemokine ligand 25			-2.62	0.0001		
CCR9	C-C motif chemokine receptor 9			5.09	0.0249		
CD28	CD28 molecule			-3.16	0.0001		
CD40	CD40 molecule			2.38	0.0001		
CXCL12	C-X-C motif chemokine ligand 12			-2.85	0.0001		
CXCR4	C-X-C motif chemokine receptor 4			2.09	0.0007		
ICOSLG	Inducible T cell costimulator ligand			-2.36	0.0001		
ITGB7	Integrin subunit beta 7			-2.27	0.0001		
PIGR	Polymeric immunoglobulin receptor			2.37	0.0001		
TGFB1	Transforming growth factor beta 1			2.02	0.0001		
TNFRSF13B	TNF receptor superfamily member 13b			-2.57	0.0003		
TNFSF13	TNF superfamily member 13			3.47	0.0310		

Fig S1. Visualization of qualities of sequencing raw data. (A) Throughput of total raw data; (B) total read count of raw data; (C) GC/AT content of raw data; (D) Q20/Q30 scores of raw data

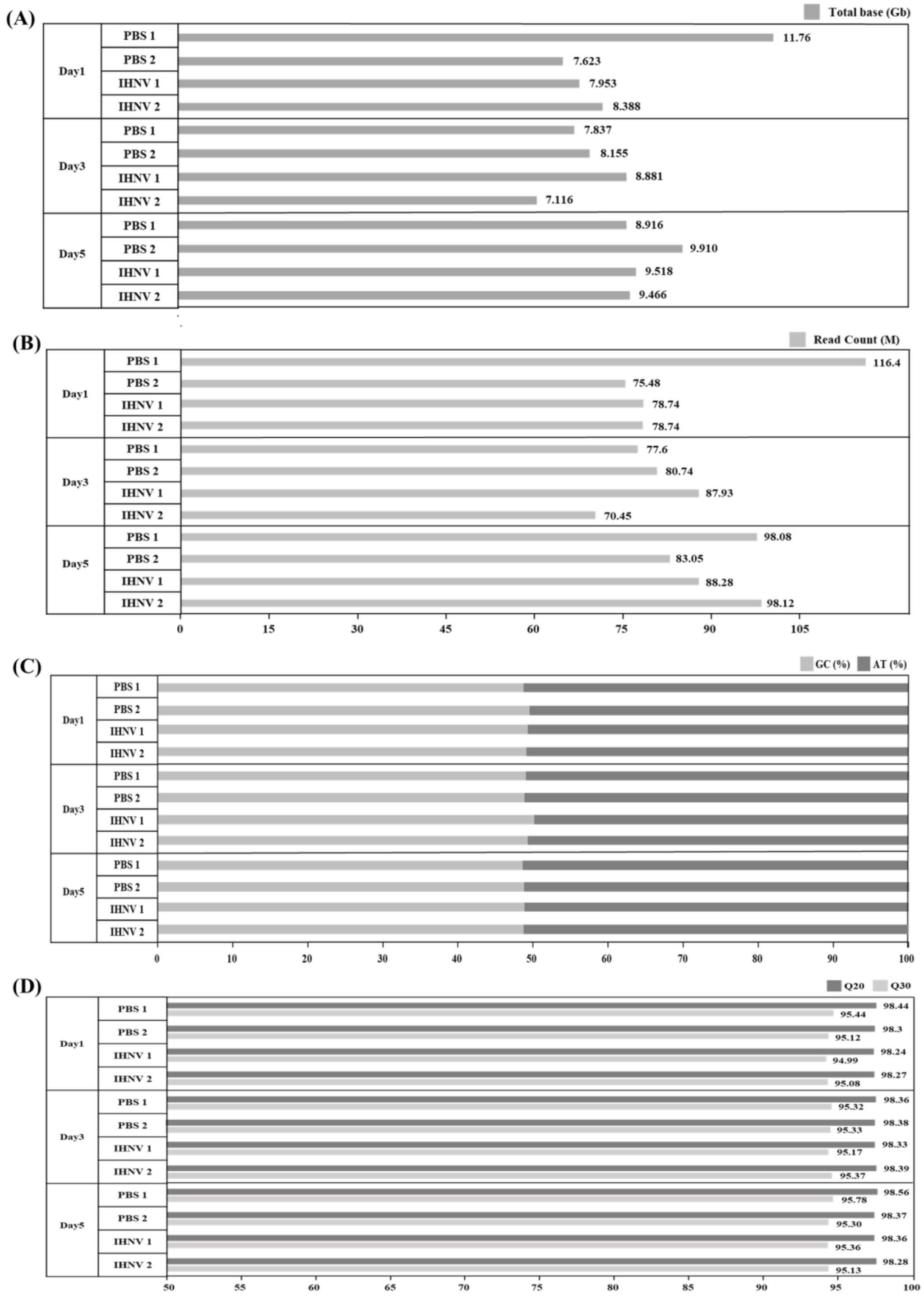

Fig S2. Quality assessment and comparison of transcriptome data quality between control and IHNV groups. A) Correlation matrix of the transcriptome data of all samples. B) Summary of the differentially expressed genes in the control and IHNV groups. (C) Correlation analysis of RT-qPCR and RNA-seq. Correlation of fold change analyzed by data obtained using RT-qPCR (x-axis) with RNA-seq platform (y-axis).

(A)

		Day 1				Day 3				Day 5			
		P1	P2	I1	I2	P1	P2	I1	I2	P1	P2	I1	I2
Day 1	PBS 1	1	0.958	0.919	0.91	0.959	0.955	0.756	0.783	0.906	0.938	0.854	0.842
	PBS 2	0.958	1	0.93	0.917	0.967	0.966	0.785	0.79	0.954	0.965	0.893	0.883
	IHNV 1	0.919	0.93	1	0.925	0.913	0.935	0.825	0.842	0.908	0.92	0.91	0.88
	IHNV 2	0.91	0.917	0.925	1	0.91	0.923	0.805	0.809	0.9	0.919	0.9	0.891
Day 3	PBS 1	0.959	0.967	0.913	0.91	1	0.968	0.731	0.741	0.943	0.962	0.849	0.841
	PBS 2	0.955	0.966	0.935	0.923	0.968	1	0.755	0.767	0.937	0.966	0.866	0.856
	IHNV 1	0.756	0.785	0.825	0.805	0.731	0.755	1	0.954	0.771	0.767	0.901	0.906
	IHNV 2	0.783	0.79	0.842	0.809	0.741	0.767	0.954	1	0.776	0.779	0.902	0.901
Day 5	PBS 1	0.906	0.954	0.908	0.9	0.943	0.937	0.771	0.776	1	0.961	0.888	0.878
	PBS 2	0.938	0.965	0.92	0.919	0.962	0.966	0.767	0.779	0.961	1	0.886	0.878
	IHNV 1	0.854	0.893	0.91	0.9	0.849	0.966	0.901	0.902	0.888	0.886	1	0.97
	IHNV 2	0.842	0.883	0.88	0.891	0.841	0.856	0.906	0.901	0.878	0.878	0.97	1

R^2
1.0~0.92
0.92~0.80
0.80~0.70
0.70~0.00

(B)

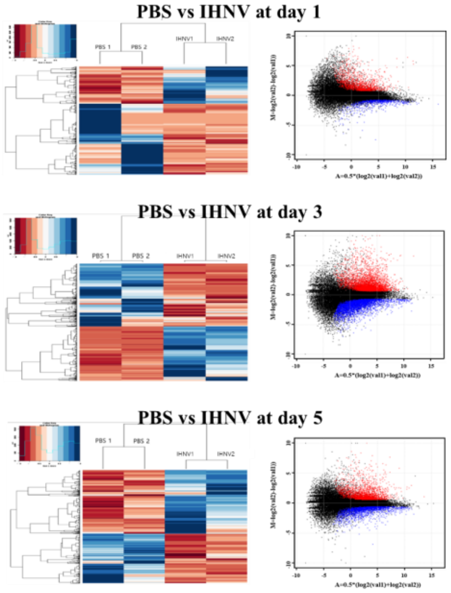

(C)

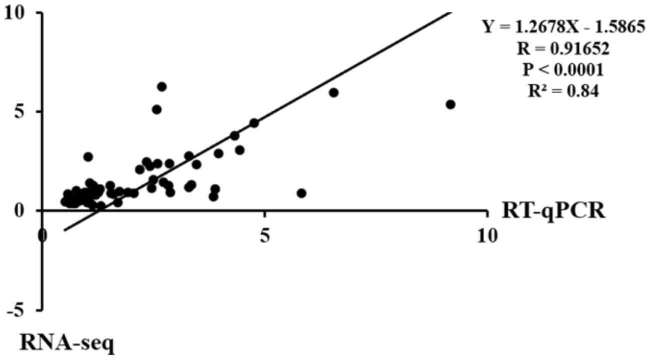

Supplement: Supplementary file 1 [file viruses-14-00859-s001.zip › viruses-1657621-supplementary.pdf]
